# Engineering Metastability in Atomic Layer Deposition: Polymorph and Valence Control

**DOI:** 10.1002/smll.202511297

**Published:** 2026-01-25

**Authors:** Jihoon Jeon, Seung Ho Ryu, Seungwan Ye, Gwang Min Park, Seong Keun Kim

**Affiliations:** ^1^ Electronic and Hybrid Materials Research Center Korea Institute of Science and Technology Seoul Republic of Korea; ^2^ KU‐KIST Graduate School of Converging Science and Technology Korea University Seoul Republic of Korea

**Keywords:** atomic layer deposition, metastable phase, polymorphism, valence control

## Abstract

Metastable phases characterized by their higher‐energy states offer promising functionalities for electronic, catalytic, and energy applications. However, their synthesis is often hindered by high formation energy barriers and thermodynamic constraints. Atomic layer deposition (ALD) has attracted significant interest for a wide range of applications, including semiconductors, advanced electronics, and energy‐related applications, owing to its exceptional features, including low‐temperature processing, precise atomic‐scale control, and excellent conformality. Despite these advantages, the inherently low thermal budget of ALD poses significant challenges for the synthesis of metastable phases. This review presents a comprehensive overview of the recent advances in the engineering of metastability via ALD. This review categorizes the manifestations of metastability in ALD into two main directions: polymorphic transformations and valence state control. For polymorphs, strategies, such as temperature modulation, substrate‐induced lattice matching, grain‐size refinement, doping, and solid‐solution formation, enable selective phase stabilization. Approaches for valence control include temperature modulation, the design and selection of the precursor/reactant, and post‐deposition treatments. By linking reaction mechanisms with material phases, this review offers insights into the stabilization of metastable phases and practical design principles for achieving them. These insights will pave the way for new functional materials that surpass conventional thermodynamic limitations and advance next‐generation devices and technologies.

## Graphical abstract

This work presents strategies for stabilizing two types of metastable phases: structural polymorphs and multivalence states via ALD. These approaches focus on lowering the energy barriers required for metastable phase formation, enabling access to metastable states beyond the thermodynamic limits imposed by the low‐temperature conditions of ALD. This review offers practical guidance for achieving controlled phase formation in ALD‐grown films.

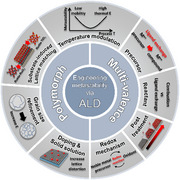

## 1 Introduction

Advances in modern technologies are approaching the performance limits of conventional materials, thereby increasing the demand for new functional materials with enhanced properties and greater versatility. Metastable phases are commonly defined as states that reside at higher free energy than the thermodynamic ground state at a given composition and temperature. In many oxide, nitride, and chalcogenide systems, metastable polymorphs lie within a moderate energy offset—typically on the order of tens of meV per atom—and may be experimentally accessible if kinetic barriers prevent relaxation into the equilibrium phase [1]. In practical film processing, it is also necessary to consider states that behave effectively as metastable within the accessible temperature window. These are phases that do not appear as equilibrium phases under those conditions because the system is kinetically trapped before reaching the ground state or distributed among several closely competing configurations, such as multiple oxidation states of transition‐metal cations. These hard‐to‐form phases differ from conventionally used stable phases in their atomic coordination and bond lengths, and these deviations often translate into superior performance. Metastable phases have been used in various applications, including emerging semiconductors [2, 3, 4, 5, 6, 7, 8, 9], nanoelectronics [10, 11, 12, 13, 14, 15, 16], optoelectronics [17, 18, 19, 20, 21], catalysts [22, 23, 24, 25, 26], sensors [27, 28, 29, 30], and energy storage systems [31, 32, 33, 34, 35]. This suggests that metastable phases can create new opportunities for material technologies.

Despite their potential, the design and synthesis of metastable phases remain challenging. Their formation typically requires bypassing thermodynamically favorable pathways and overcoming substantial energy barriers, often necessitating extreme conditions, such as high temperatures or pressures. However, these conditions are not readily compatible with conventional thin‐film fabrication processes.

Atomic layer deposition (ALD) is a thin‐film growth technique based on self‐limiting surface reactions between precursors and functional groups on the reaction surface. Owing to this self‐limiting mechanism, ALD offers precise thickness control at the atomic level with outstanding uniformity and conformality over complex 3D structures [36]. These unique advantages have made ALD a critical tool for a wide range of applications, including advanced electronics [37, 38, 39, 40, 41, 42, 43, 44], energy storage [45, 46, 47, 48, 49, 50], energy conversion [51, 52, 53, 54, 55], and catalysis [56, 57, 58, 59]. However, the self‐limiting mechanism that enables these advantages also imposes constraints; it is effective only below the thermal decomposition temperature of the precursors, limiting most ALD processes to temperatures typically below 350°C [60, 61], which is significantly lower than the temperatures used in sputtering or chemical vapor deposition. This inherently low thermal budget imposes kinetic limitations on atomic rearrangement and long‐range diffusion, preventing atoms from overcoming the thermodynamic barriers required to form metastable phases. Moreover, the sequential, cycle‐by‐cycle nature of ALD suppresses non‐equilibrium pathways that are often critical in other high‐energy deposition techniques. Consequently, both the narrow ALD temperature window and its self‐limiting reaction mechanism pose significant challenges for the formation of metastable phases. Therefore, ALD may be less favorable than other growth techniques for the realization of metastable phases.

**FIGURE 1:**
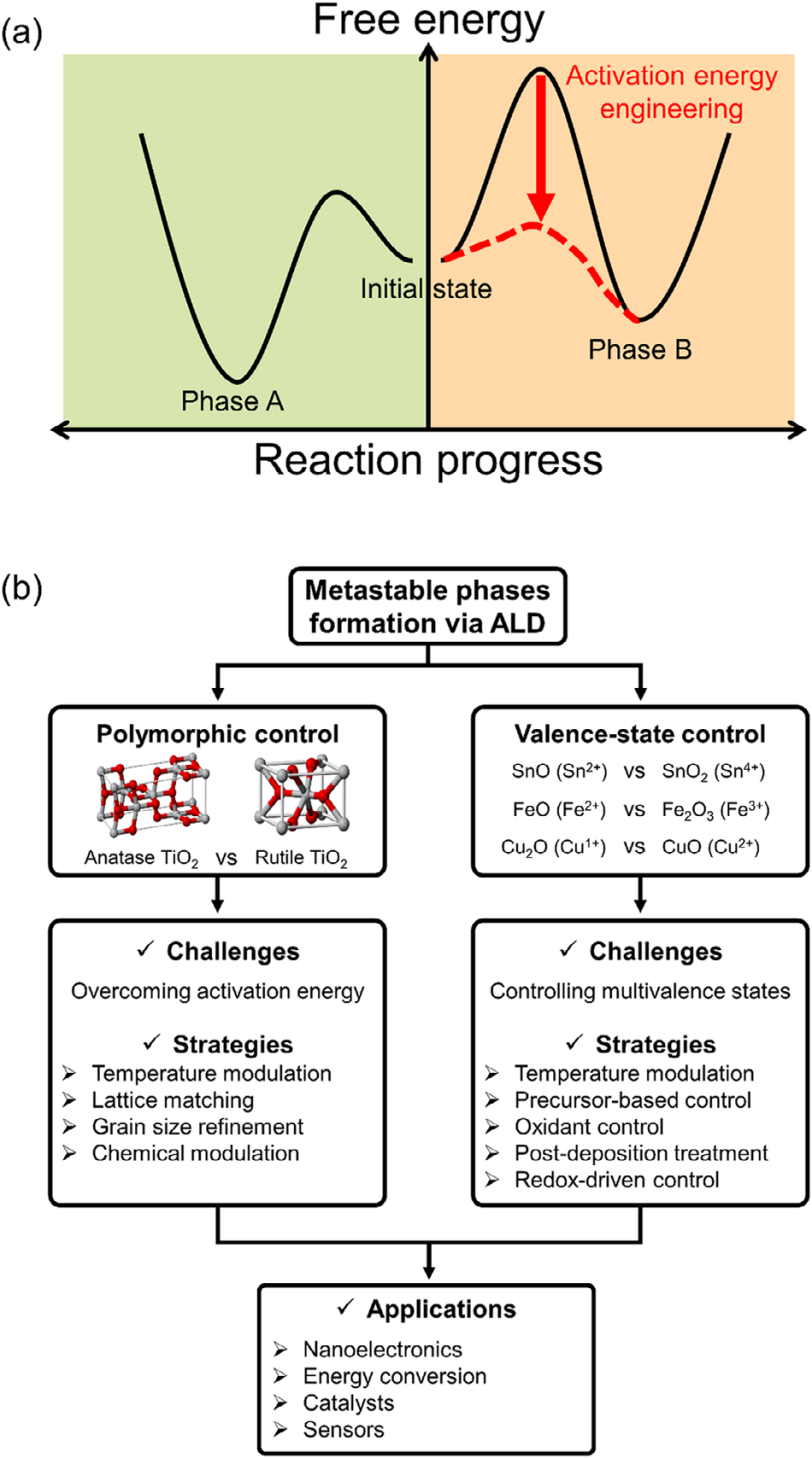
(a) Schematic of the engineering of the activation barrier for the selection of metastable phases. (b) Overview of ALD strategies for synthesizing metastable polymorphic and multivalent phases, along with their respective challenges.

This apparent contradiction raises a fundamental question: Can metastable phases be synthesized via ALD, and if so, what strategies enable their formation? As illustrated in Figure 1a, the solution lies in strategies used to engineer the activation barrier associated with the formation of metastable phases. Recent studies have demonstrated that the synthesis of certain metastable phases via ALD is feasible through the precise control of the process conditions. Furthermore, ALD offers several tools, such as surface chemistry engineering, interface‐mediated templating, and nanoscale confinement, which can be exploited to stabilize metastable phases beyond conventional thermodynamic limits.

In this review, we discuss the strategies for stabilizing metastable phases in thin films using ALD, with a particular focus on phases that are thermodynamically or effectively metastable under the conventional ALD temperature window, yet can be realized and retained through appropriate process design. Before introducing these strategies, we classified the types of metastability observed in ALD‐grown films into two main classes. The first involves controlling the crystal structure, where the materials exhibit polymorphism, that is, the ability to form different crystal structures (such as anatase vs. rutile TiO_2_ or monoclinic vs. orthorhombic HfO_2_) depending on the reaction pathway. The second focuses on controlling the valence state, in which transition metal cations (e.g., Fe^2+^/Fe^3+^ and Sn^2+^/Sn^4+^) can exist in multiple valence states, some of which are metastable. Figure 1b summarizes this classification and outlines the major challenges and representative strategies associated with each direction. For polymorphic control, the main issue lies in overcoming the activation energy barrier under the low‐temperature constraints of ALD. The challenge of valence state control originates from the maintenance of unstable states under typical ALD conditions.

Through this framework, we aim to clarify how metastable phases can be accessed and stabilized during the ALD process, emphasizing that understanding this control is essential for enabling the next generation of high‐performance functional materials.

## 2 Strategies for Realizing Metastable Polymorphs via ALD

In polymorphic materials, there is only one crystal structure with a thermodynamic ground state, whereas other polymorphs possess higher formation energies. However, certain metastable phases with formation energies within the metastability range (<approximately 70 meV atom^−1^ above the ground state) [1] can be readily synthesized and observed. Notably, a large number of such polymorphic metastable phases have been successfully synthesized via ALD despite its low‐temperature limits. Representative examples are listed in Table 1 [62, 63, 64, 65, 66, 67, 68, 69, 70, 71, 72, 73, 74, 75, 76, 77, 78, 79, 80, 81, 82, 83, 84, 85, 86, 87, 88, 89, 90, 91, 92, 93, 94, 95, 96, 97, 98, 99, 100, 101, 102, 103, 104, 105, 106, 107, 108, 109, 110, 111, 112, 113, 114, 115, 116, 117, 118, 119, 120, 121, 122, 123, 124, 125, 126, 127, 128, 129, 130, 131, 132, 133, 134, 135, 136, 137, 138, 139, 140, 141, 142, 143, 144, 145, 146, 147, 148, 149, 150, 151, 152, 153, 154, 155, 156, 157, 158, 159, 160, 161, 162, 163, 164, 165, 166, 167, 168, 169, 170, 171, 172, 173, 174, 175, 176, 177, 178, 179, 180, 181].

**TABLE 1:** Overview of ALD processes for metastable polymorphic phases. This table summarizes, for each material, the precursor/reactant combinations, process windows, substrates, and heteroelements used to stabilize the corresponding metastable polymorph.

Element	Metastable polymorph	Precursor	Reactant	Substrate	Hetero‐element	Growth temperature (°C)	References
Be	Rocksalt‐BeO	BeMe_2_	H_2_O	Si	Mg	250	[62]
Ti	Rutile‐TiO_2_	TiCl_4_	H_2_O	Si, SiO_2_	—	425‐600	[63]
	Rutile‐TiO_2_	TiCl_4_	H_2_O	RuO_2_	—	400	[64, 65, 66]
	Rutile‐TiO_2_	TiCl_4_	H_2_O	C‐cut sapphire	—	600	[67]
	Rutile‐TiO_2_	TiCl_4_	O_3_	Si, SiO_2_	—	600	[68]
	Rutile‐TiO_2_	TiCl_4_	O_3_	RuO_2_	—	250‐450	[69, 70, 71]
	Rutile‐TiO_2_	TiCl_4_	O_3_	R‐cut sapphire	—	350	[72]
	Rutile‐TiO_2_	TiI_4_	H_2_O	SiO_2_	—	445	[73]
	Rutile‐TiO_2_	TiI_4_	H_2_O_2_	Si, SiO_2_	—	455	[74, 75]
	Rutile‐TiO_2_	TiI_4_	H_2_O_2_	R,C‐cut sapphire	—	275‐375	[74]
	Rutile‐TiO_2_	TiI_4_	H_2_O_2_	MgO(001)	—	455	[74]
	Rutile‐TiO_2_	TiI_4_	O_2_	Si	—	457	[76]
	Rutile‐TiO_2_	Ti(O^i^Pr)_4_	H_2_O	Si	Nb	300	[77]
	Rutile‐TiO_2_	Ti(O^i^Pr)_4_	H_2_O	RuO_2_	—	250	[78]
	Rutile‐TiO_2_	Ti(O^i^Pr)_4_	O_2_ plasma	Ru	—	250	[79]
	Rutile‐TiO_2_	Ti(O^i^Pr)_4_	O_2_ plasma	RuO_2_	—	250	[80]
	Rutile‐TiO_2_	Ti(O^i^Pr)_4_	O_3_	Ru, Ir	—	250	[81, 82, 83, 84, 85, 86]
	Rutile‐TiO_2_	Ti(O^i^Pr)_4_	O_3_	RuO_2_, SnO_2_, MoO_2_	—	250	[87, 88, 89]
	Rutile‐TiO_2_	Ti(CpMe_5_)(OMe)_3_	O_2_ plasma	TiN	Sn	350	[90]
	Rutile‐TiO_2_	Ti(CpMe_5_)(OMe)_3_	O_3_	TiN	Sn	350	[91]
	Rutile‐TiO_2_	Ti(CpMe_5_)(OMe)_3_	O_3_	Ru	—	330	[92]
	Rutile‐TiO_2_	Ti(CpMe_5_)(OMe)_3_	O_3_	RuO_2_, MoO_2_	—	300‐350	[93, 94, 95, 96, 97]
	Rutile‐TiO_2_	Ti(OEt)_4_	H_2_O	Si	—	350	[98]
	Rutile‐TiO_2_	Ti(NMe_2_)_4_	H_2_O	Si	—	200	[99]
	TiO_2_‐II	TiCl_4_	H_2_O	Si	—	425	[100, 101]
	TiO_2_‐II	TiCl_4_	H_2_O	C‐cut sapphire	—	425	[67]
	TiO_2_‐II	TiCl_4_	O_3_	C‐cut sapphire	—	300‐450	[69, 72]
Mn	ε‐MnO_2_	Mn(thd)_3_	O_3_	C‐cut sapphire	—	186	[102]
Fe	β‐Fe_2_O_3_	Fe(Cp)_2_	O_3_	ITO	—	200	[103]
Ga	α‐Ga_2_O_3_	GaI_3_	O_3_	α‐Cr_2_O_3_	—	275‐550	[104, 105]
	α‐Ga_2_O_3_	GaMe_3_	O_2_ plasma	C‐cut sapphire	—	250‐295	[106, 107, 108]
	κ/ε‐Ga_2_O_3_	GaI_3_	O_3_	Si, SiO_2_	—	450‐550	[104, 105]
	κ/ε‐Ga_2_O_3_	GaMe_3_	O_2_ plasma	C‐cut sapphire	—	365	[106]
Zr	Fluorite‐ZrO_2_	ZrCl_4_	H_2_O	Si	—	210‐600	[174, 175, 179]
	Fluorite‐ZrO_2_	ZrI_4_	H_2_O_2_	Si	—	250‐375	[176, 178]
	Fluorite‐ZrO_2_	CpZr(NMe_2_)_3_	H_2_O	Si	—	200‐400	[177]
	Fluorite‐ZrO_2_	Zr(NEtMe)_4_	O_3_	TiN	—	225‐300	[180, 181]
Mo	Rutile‐MoO_2_	Unknown	O_3_	TiN	Sn	300	[97]
	β‐MoO_3_	Mo(CO)_6_	O_2_ plasma	SiO_2_	—	160	[109]
	β‐MoO_3_	Mo(CO)_6_	O_3_	Si	—	165	[110]
	β‐MoO_3_	Mo(CO)_6_	Mo(CO)_6_	C‐cut sapphire	—	167	[111]
In	α‐In_2_O_3_	In(DMAE‐^t^Bu)	H_2_O	Si	—	100‐150	[112]
Sn	π‐SnS	Sn(DPFA)_2_	H_2_S	Si, SiO_2_	—	80‐120	[113, 114]
	π‐SnS	Sn(dmamp)_2_	H_2_S	Si, SiO_2_	—	90‐150	[115]
	π‐SnS	Sn(acac)_2_	H_2_S	Si, Al_2_O_3_	—	80‐120	[116, 117]
	π‐SnS	Sn(Bu)_2_(diamido)	H_2_S	SiO_2_	—	25‐100	[118]
Hf	Fluorite‐HfO_2_	HfCl_4_	H_2_O	Si	—	300‐940	[119, 120, 121, 122, 123, 124, 125, 126]
	Fluorite‐HfO_2_	HfCl_4_	H_2_O	TiN	Al	300	[127]
	Fluorite‐HfO_2_	HfCl_4_	H_2_O	TiN	Sr	300	[128]
	Fluorite‐HfO_2_	HfCl_4_	H_2_O	Si, TiN	Gd	300	[129, 130]
	Fluorite‐HfO_2_	HfCl_4_	O_3_	Si	—	450‐600	[131]
	Fluorite‐HfO_2_	HfCl_4_	O_3_	Si	Pr	325	[132, 133]
	Fluorite‐HfO_2_	HfI_4_	H_2_O	Si	—	600	[122]
	Fluorite‐HfO_2_	Hf(NMe_2_)_4_	H_2_O	Si	Zr	250	[134]
	Fluorite‐HfO_2_	Hf(NMe_2_)_4_	O_2_ plasma	W	Ga	270	[135]
	Fluorite‐HfO_2_	Hf(NMe_2_)_4_	O_3_	TiN	Zr	250‐285	[136, 137, 138, 139]
	Fluorite‐HfO_2_	Hf(NEtMe)_4_	H_2_O	TiN	Al	200	[140]
	Fluorite‐HfO_2_	Hf(NEtMe)_4_	H_2_O	TiN	Si	200‐300	[127, 140]
	Fluorite‐HfO_2_	Hf(NEtMe)_4_	H_2_O	TiN	V	240	[141]
	Fluorite‐HfO_2_	Hf(NEtMe)_4_	H_2_O	TiN	Zr	250	[142]
	Fluorite‐HfO_2_	Hf(NEtMe)_4_	H_2_O	TiN	Zr	260	[143]
	Fluorite‐HfO_2_	Hf(NEtMe)_4_	H_2_O	TiN	Zr	250‐300	[144]
	Fluorite‐HfO_2_	Hf(NEtMe)_4_	O_2_	Si	—	280	[145]
	Fluorite‐HfO_2_	Hf(NEtMe)_4_	O_2_ plasma	Si	Al	250	[146]
	Fluorite‐HfO_2_	Hf(NEtMe)_4_	O_2_ plasma	Si, TaN	Si	200	[147, 148]
	Fluorite‐HfO_2_	Hf(NEtMe)_4_	O_2_ plasma	TiN	La	235	[149]
	Fluorite‐HfO_2_	Hf(NEtMe)_4_	O_3_	TiN	—	200‐220	[150]
	Fluorite‐HfO_2_	Hf(NEtMe)_4_	O_3_	TiN	Al	300	[151]
	Fluorite‐HfO_2_	Hf(NEtMe)_4_	O_3_	TiN	Y	275‐300	[152, 153]
	Fluorite‐HfO_2_	Hf(NEtMe)_4_	O_3_	TiN	Zr	260	[143]
	Fluorite‐HfO_2_	Hf(NEtMe)_4_	O_3_	TiN	Zr	280	[154, 155, 156]
	Fluorite‐HfO_2_	Hf(NEtMe)_4_	O_3_	TiN	La	250	[157]
	Fluorite‐HfO_2_	Hf(NEtMe)_4_	O_3_	TiN	La	265	[158]
	Fluorite‐HfO_2_	Hf(NEtMe)_4_	O_3_	TaN	La	300	[159]
	Fluorite‐HfO_2_	Hf(O^t^Bu)(NEtMe)_3_	O_3_	Rutile‐TiO_2_	—	250	[160]
	Fluorite‐HfO_2_	HfCp(NMe_2_)_3_	O_3_	TiN	Y	300	[161]
	Fluorite‐HfO_2_	HfCp(NMe_2_)_3_	O_3_	TiN	Zr	300‐320	[162, 163, 164]
	Fluorite‐HfO_2_	Hf(CpMe)_2_(OMe)Me	O_3_	TiN	Y	300‐350	[152]
Ta	δ‐Ta_2_O_5_	TaF_5_	H_2_O	Si	—	400‐450	[165]
	δ‐Ta_2_O_5_	TaCl_5_	H_2_O	Si, SiO_2_	—	300‐500	[166, 167]
	δ‐Ta_2_O_5_	TaI_5_	H_2_O_2_	Si	—	400	[168]
	δ‐Ta_2_O_5_	Ta(OEt)_5_	H_2_O, O_3_	TiN	—	300	[169]
Bi	β‐Bi_2_O_3_	Bi(OCMe_2_^i^Pr)	H_2_O	TaN, TiN, Si_3_N_4_	—	90‐210	[170]
	β‐Bi_2_O_3_	Bi(ph)_3_	O_3_	LAO(001)	—	280	[171]
	β‐Bi_2_O_3_	Bi(ph)_3_	O_3_	STO(001)	—	280	[171]
	β‐Bi_2_O_3_	Bi(mmp)_3_	O_3_	STO(001)	Fe	200	[172]
	γ‐Bi_2_O_3_	Bi(thd)_3_	H_2_O	Si	—	200‐300	[173]
	γ‐Bi_2_O_3_	Bi(ph)_3_	O_3_	Si	—	280	[171]
	δ‐Bi_2_O_3_	Bi(mmp)_3_	O_3_	YSZ(111)	Fe	200	[172]

The significance of synthesizing metastable polymorphs via ALD extends far beyond academic interest. Several of these phases demonstrated practical utility in advanced technological applications. A representative example is tetragonal ZrO_2_, which is used as a high‐k dielectric in commercial dynamic random‐access memory (DRAM) capacitors. While the stable monoclinic phase of ZrO_2_ exhibits a low dielectric constant of approximately 15 [182], the ALD‐grown tetragonal phase achieves a significantly high dielectric constant of ∼ 40 [181, 183], enabling the further scaling of DRAM cells. In addition, the orthorhombic phase of HfO_2_, a metastable phase exhibiting ferroelectricity, has attracted considerable attention for use in next‐generation memory and logic devices [142, 184, 185, 186, 187].

The successful formation of metastable phases via ALD cannot be attributed to their low relative formation energies. Instead, phase‐selective mechanisms are essential for guiding stabilization. In the following subsections, we explore the primary strategies that enable phase selectivity in ALD‐grown films: the control of the deposition temperature, substrate‐induced lattice matching, grain‐size refinement, and chemical modulation via doping or solid‐solution formation.

### 2.1 Control of Deposition Temperature

**FIGURE 2:**
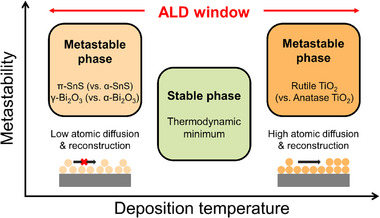
Schematic of the temperature‐dependent engineering of metastable phases in ALD.

Temperature and pressure are among the most fundamental and effective parameters for selectively controlling the crystal structures of polymorphic materials. However, owing to the intrinsic characteristics of ALD, applying high pressure is practically unfeasible. Moreover, as described in the Introduction, the deposition temperatures in most ALD processes are limited to below approximately 350°C. Nevertheless, even when using the same precursor, the target material may exhibit multiple crystal structures, depending on the specific reaction pathways available. If the energy barriers of these pathways are similar, the resulting crystal structure can vary within the ALD window, as shown in Figure 2. In some materials, low atomic mobility favors the formation of metastable structures even at low temperatures, whereas in others, an increased deposition temperature can provide sufficient thermal energy to induce phase transitions into metastable polymorphs. Representative examples of the temperature‐dependent formation of metastable phases are summarized in Table 2 [63, 68, 73, 74, 75, 76, 98, 99, 100, 101, 104, 105, 109, 110, 111, 112, 113, 114, 115, 116, 117, 118, 119, 120, 121, 122, 123, 124, 125, 126, 131, 165, 166, 167, 168, 169, 170, 171, 173, 188].

**TABLE 2:** Representative examples of ALD processes for the formation of metastable polymorphs via temperature modulation. This table summarizes, for each precursor/reactant combination, the growth and annealing temperatures at which metastable and stable phases appear, revealing the temperature window for metastable phase formation and whether it lies below or above that of the stable polymorph.

Element	Metastable polymorph	Precursor	Reactant	Growth temperature (°C)	Annealing temperature (°C)	Temperature window for stable phases (°C)	References
Ti	Rutile‐TiO_2_	TiCl_4_	H_2_O	425‐600	—	165‐400(Anatase)	[63, 68, 98, 100, 101, 188]
	Rutile‐TiO_2_	TiCl_4_	O_3_	600	—	250‐500(Anatase)	[68]
	Rutile‐TiO_2_	TiI_4_	H_2_O	445	—	135‐375(Anatase)	[73]
	Rutile‐TiO_2_	TiI_4_	H_2_O_2_	455	—	230‐375(Anatase)	[74, 75]
	Rutile‐TiO_2_	TiI_4_	O_2_	457	—	235‐380(Anatase)	[76]
	Rutile‐TiO_2_	Ti(OEt)_4_	H_2_O	350	750	350(Anatase)	[98]
	Rutile‐TiO_2_	Ti(NMe_2_)_4_	H_2_O	200	500	375‐500(Anatase)	[99]
	TiO_2_‐II	TiCl_4_	H_2_O	425	—	165‐350(Anatase)	[100, 101]
Ga	κ/ε‐Ga_2_O_3_	GaI_3_	O_3_	450‐550	—	425(Amorphous)	[104, 105]
Mo	β‐MoO_3_	Mo(CO)_6_	O_2_ plasma	160	400	500‐600(α‐MoO_3_)	[109]
	β‐MoO_3_	Mo(CO)_6_	O_3_	165	300	400‐600(α‐MoO_3_)	[110, 111]
In	α‐In_2_O_3_	In(DMAE‐^t^Bu)	H_2_O	100‐150	—	200‐250(Bixbyite)	[112]
Sn	π‐SnS	Sn(DPFA)_2_	H_2_S	80‐120	—	140‐200(α‐SnS)	[113, 114]
	π‐SnS	Sn(dmamp)_2_	H_2_S	90‐150	—	180‐240(α‐SnS)	[115]
	π‐SnS	Sn(acac)_2_	H_2_S	80‐120	—	175(α‐SnS)	[116, 117]
	π‐SnS	Sn(Bu)_2_(diamido)	H_2_S	25‐100	—	125‐250(α‐SnS)	[118]
Hf	Fluorite‐HfO_2_	HfCl_4_	H_2_O	300‐940	—	226‐600(Monoclinic)	[119, 120, 121, 122, 123, 124, 125, 126]
	Fluorite‐HfO_2_	HfCl_4_	O_3_	450‐600	—	225‐350(Monoclinic)	[131]
	Fluorite‐HfO_2_	HfI_4_	H_2_O	600	—	300‐450(Monoclinic)	[122]
Ta	δ‐Ta_2_O_5_	TaF_5_	H_2_O	400‐450	—	—	[165]
	δ‐Ta_2_O_5_	TaCl_5_	H_2_O	300‐500	—	400(β‐Ta_2_O_5_)	[166, 167]
	δ‐Ta_2_O_5_	TaI_5_	H_2_O_2_	400	—	350(β‐Ta_2_O_5_)	[168]
	δ‐Ta_2_O_5_	Ta(OEt)_5_	H_2_O, O_3_	300	—	—	[169]
Bi	β‐Bi_2_O_3_	Bi(OCMe_2_^i^Pr)	H_2_O	90‐210	—	800(α‐Bi_2_O_3_)	[170]
	γ‐Bi_2_O_3_	Bi(thd)_3_	H_2_O	200‐300	600‐700	300‐500(α‐Bi_2_O_3_)	[173]
	γ‐Bi_2_O_3_	Bi(ph)_3_	O_3_	280	500‐700	400(α‐Bi_2_O_3_)	[171]

These examples, such as the formation of metastable π‐SnS (vs. α‐SnS), β‐Bi_2_O_3_ and γ‐Bi_2_O_3_ (vs. α‐Bi_2_O_3_) at low deposition temperatures, highlight the role of kinetic constraints in ALD. At low temperatures, limited atomic diffusion and insufficient surface reconstruction can induce the nucleation of metastable polymorphs during the early growth stages. SnS is a typical example of the phenomenon. In ALD processes using various Sn precursors and H_2_S gas, the ALD process at below approximately 150°C favors the formation of the thermodynamically metastable π‐phase (Cubic, Pa‐3), whereas the thermodynamically stable α‐phase (Orthorhombic, Pnma) becomes dominant as the temperature increases [113, 114, 115, 116, 117, 118]. A similar trend was observed in the ALD of In_2_O_3_ films. In an ALD process employing In(DMAE‐tBu) and H_2_O, the metastable rhombohedral phase (R‐3c) is favored at temperatures below ∼160°C, whereas the thermodynamically stable cubic phase (Ia‐3) dominates at higher temperatures [112]. These examples demonstrate that carefully controlled low‐temperature ALD conditions selectively promote the formation of metastable crystal structures.

In contrast, certain metastable phases were formed at elevated deposition temperatures. Notably, unlike metal–organic precursors, halide precursors extend the ALD temperature window to temperatures far exceeding 400°C, thereby enabling the stabilization of certain metastable phases. TiO_2_ is typically deposited in the thermodynamically stable anatase phase (Tetragonal, I4_1_/amd) under most ALD conditions. However, when halide precursors, such as TiCl_4_ or TiI_4_, are employed and the deposition temperatures exceed 600°C, the partial formation of the rutile phase (Tetragonal, P4_2_/mnm) has been reported [63, 68, 73, 74, 75, 76, 98, 100, 101, 188]. In addition, the TiO_2_‐II phase (Orthorhombic, Pbcn), which is typically stabilized only under high‐pressure conditions, has been reported to form at elevated temperatures [100, 101]. Similar phenomena have been observed in ALD‐grown HfO_2_ [119, 120, 121, 122, 123, 124, 125, 126, 131], Ta_2_O_5_ [165, 166, 167, 168], and Ga_2_O_3_ [104, 105], where the use of halide precursors enables high‐temperature growth that facilitates the formation of metastable polymorphs not observed within the conventional ALD window.

Post‐deposition annealing (PDA) is an effective strategy to stabilize metastable polymorphs. For instance, amorphous MoO_3_ films deposited by ALD at temperatures below 165°C using Mo(CO)_6_ are transformed into the metastable β‐phase (Monoclinic, P2_1_/c) upon annealing at approximately 300°C [109, 110, 111]. Further annealing above 400°C leads to predominant crystallization into the thermodynamically stable α‐phase (Orthorhombic, Pnma). Similarly, Bi_2_O_3_ films initially deposited in the stable α‐phase (Monoclinic, P2_1_/c) at 250–300°C using Bi(thd)_3_ or Bi(ph)_3_ can be recrystallized into the γ‐phase (Cubic, I23) through PDA at temperatures above 500°C [171, 173]. These examples suggest that the thermal energy supplied through PDA can effectively overcome kinetic barriers, enabling the formation of metastable phases.

### 2.2 Substrate‐Induced Lattice Matching

**FIGURE 3:**
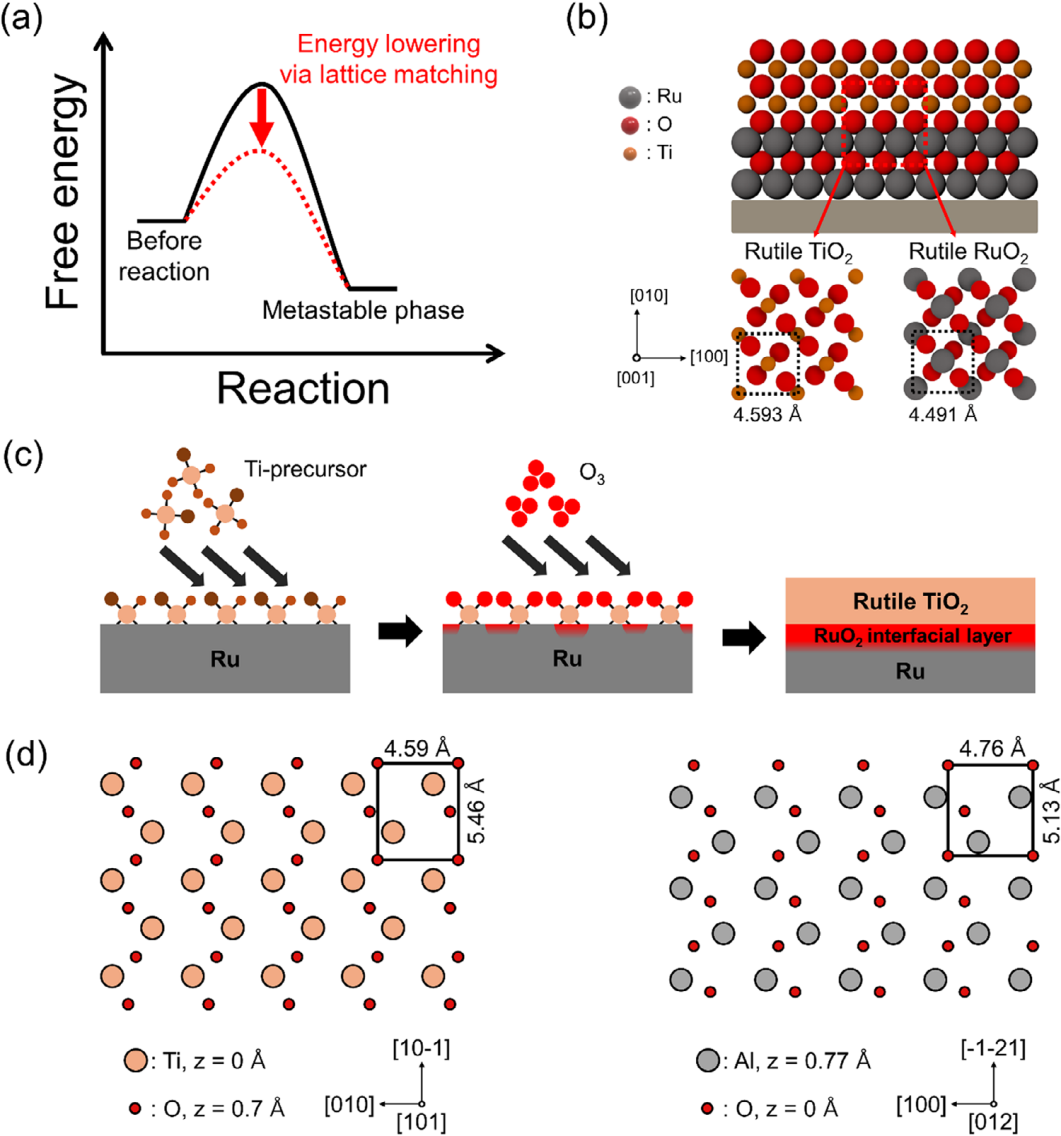
Substrate‐induced pathways for stabilizing metastable phases via lattice or domain matching. (a) Schematic for lowering the activation barrier for stabilizing metastable phases via lattice‐matching. (b) Stabilization of metastable rutile TiO_2_ on RuO_2_ utilizing the structural similarity between rutile TiO_2_ and rutile RuO_2_. (c) Schematic of the formation of metastable rutile TiO_2_ on Ru via the in situ formation of a rutile RuO_2_ interfacial layer. (d) Schematic of the atomic arrangements of rutile TiO_2_ (101) and α‐sapphire (012) surfaces.

Another effective approach for stabilizing metastable polymorphs is to use substrates that provide structural compatibility with the desired phase. In particular, selecting a substrate with a similar lattice structure or matching crystallographic orientation can reduce the energy required for atomic rearrangement, thereby enabling the formation of metastable phases (Figure 3a). This strategy serves as a useful platform for selectively stabilizing metastable polymorphs while suppressing their transition to more stable polymorphs. Table 3 summarizes representative examples in which metastable crystal structures were synthesized via lattice matching between ALD‐grown films and the underlying substrates [64, 65, 66, 67, 69, 70, 71, 72, 74, 78, 79, 80, 81, 82, 83, 84, 85, 86, 87, 88, 89, 92, 93, 94, 95, 96, 97, 102, 103, 104, 105, 106, 107, 108, 160, 171, 172].

**TABLE 3:** Representative examples of ALD processes for the formation of metastable polymorphs through substrate‐induced lattice matching. This table summarizes cases where lattice‐matching substrates compatible with the desired metastable polymorph are employed to selectively form the target phase within the ALD temperature window.

Element	Metastable polymorph	Precursor	Reactant	Substrate	Growth temperature (°C)	References
Ti	Rutile‐TiO_2_	TiCl_4_	H_2_O	C‐cut sapphire	600	[67]
	Rutile‐TiO_2_	TiCl_4_	H_2_O	RuO_2_	400	[64, 65, 66]
	Rutile‐TiO_2_	TiCl_4_	O_3_	R‐cut sapphire	350‐450	[69, 72]
	Rutile‐TiO_2_	TiCl_4_	O_3_	RuO_2_	250‐350	[70, 71]
	Rutile‐TiO_2_	TiI_4_	H_2_O_2_	R‐cut sapphire	275	[74]
	Rutile‐TiO_2_	TiI_4_	H_2_O_2_	C‐cut sapphire	375	[74]
	Rutile‐TiO_2_	TiI_4_	H_2_O_2_	MgO(001)	455	[74]
	Rutile‐TiO_2_	Ti(O^i^Pr)_4_	H_2_O	RuO_2_	250	[78]
	Rutile‐TiO_2_	Ti(O^i^Pr)_4_	O_2_ plasma	Ru	250	[79]
	Rutile‐TiO_2_	Ti(O^i^Pr)_4_	O_2_ plasma	RuO_2_	250	[80]
	Rutile‐TiO_2_	Ti(O^i^Pr)_4_	N_2_O plasma	Ru	250	[79]
	Rutile‐TiO_2_	Ti(O^i^Pr)_4_	O_3_	Ru	250	[81, 82, 83, 84]
	Rutile‐TiO_2_	Ti(O^i^Pr)_4_	O_3_	Ir	250	[85]
	Rutile‐TiO_2_	Ti(O^i^Pr)_4_	O_3_	Ru‐Pt alloy	250	[86]
	Rutile‐TiO_2_	Ti(O^i^Pr)_4_	O_3_	RuO_2_	250	[65, 66, 80]
	Rutile‐TiO_2_	Ti(O^i^Pr)_4_	O_3_	SnO_2_	250	[87, 88]
	Rutile‐TiO_2_	Ti(O^i^Pr)_4_	O_3_	MoO_2_	250	[89]
	Rutile‐TiO_2_	Ti(CpMe_5_)(OMe)_3_	O_3_	Ru	330	[92]
	Rutile‐TiO_2_	Ti(CpMe_5_)(OMe)_3_	O_3_	RuO_2_	300‐330	[93, 94]
	Rutile‐TiO_2_	Ti(CpMe_5_)(OMe)_3_	O_3_	MoO_2_	320‐350	[95, 96, 97]
	TiO_2_‐II	TiCl_4_	H_2_O	C‐cut sapphire	425	[67]
	TiO_2_‐II	TiCl_4_	O_3_	C‐cut sapphire	300‐450	[69, 72]
Mn	ε‐MnO_2_	Mn(thd)_3_	O_3_	C‐cut sapphire	186	[102]
Fe	β‐Fe_2_O_3_	Fe(Cp)_2_	O_3_	ITO(001)	200	[103]
Ga	α‐Ga_2_O_3_	GaI_3_	O_3_	α‐Cr_2_O_3_	275‐550	[104, 105]
	α‐Ga_2_O_3_	GaMe_3_	O_2_ plasma	C‐cut sapphire	250‐295	[106, 107, 108]
	κ/ε‐Ga_2_O_3_	GaMe_3_	O_2_ plasma	C‐cut sapphire	365	[106]
Hf	Fluorite‐HfO_2_	Hf(O^t^Bu)(NEtMe)_3_	O_3_	Rutile‐TiO_2_	250	[160]
Bi	β‐Bi_2_O_3_	Bi(ph)_3_	O_3_	LAO(100), STO(100)	280	[171]
	β‐Bi_2_O_3_	Bi(mmp)_3_	O_3_	STO(100)	300‐400	[172]
	δ‐Bi_2_O_3_	Bi(mmp)_3_	O_3_	YSZ(111)	400‐650	[172]

Rutile TiO_2_ is one of the most well‐studied systems used to demonstrate this strategy. As shown in Figure 3b, rutile TiO_2_ and rutile RuO_2_ share the same crystal structure and exhibit similar lattice constants. This structural compatibility enables the selective formation of the rutile phase TiO_2_ on RuO_2_ substrates even at temperatures below 300°C [64, 65, 66, 70, 71, 78, 80, 93, 94]. Similar results have been reported for other structurally compatible substrates, such as IrO_2_ [85], SnO_2_ [87, 88], and distorted rutile‐structured MoO_2_ [89, 95, 96, 97]. Another example is Bi_2_O_3_, where deposition on a YSZ (111) substrate leads to the formation of the metastable δ‐phase (Cubic, Fm‐3m) rather than the thermodynamically stable α‐phase, at deposition temperatures above 400°C [172]. This indicates that, although the substrate‐induced lattice matching strategy facilitates phase selection, sufficient thermal energy is still required to drive the phase transformation.

This strategy can be effective even when the ALD‐grown film and underlying substrate have different crystal structures. A representative case involves utilizing the structure of an interfacial layer that forms between the film and the substrate during the ALD process. For instance, metallic substrates, such as Ru or Ir, are known to form surface oxide layers—RuO_2_ or IrO_2_—upon exposure to strongly oxidizing reactants. These oxide layers adopt a rutile structure and can serve as effective templates for the formation of rutile TiO_2_, which is a metastable phase. Consequently, the ALD of TiO_2_ on these metal substrates using strong oxidants, such as O_3_ or O_2_ plasma, promotes the formation of rutile TiO_2_ (Figure 3c) [79, 81, 82, 83, 84, 85, 92]. In addition, the selective formation of rutile TiO_2_ occurs on Ru–Pt alloy substrates when the Ru content exceeds 50% owing to the preferential formation of a RuO_2_ interfacial layer [86].

In certain cases, metastable phases can be stabilized through orientation‐specific matching even when the crystal structures of the film and substrate differ. For example, despite their different structures, rutile and α‐sapphire exhibit structural similarity between the rutile TiO_2_ (101) plane and the α‐sapphire (012) plane, as shown in Figure 3d, enabling the formation of rutile TiO_2_ at temperatures below 400°C [67, 69, 72, 74]. Similarly, domain‐matching epitaxy has been reported to stabilize other metastable structures, such as TiO_2_‐II and ε‐MnO_2_ (Hexagonal, P6_3_/mmc), on α‐sapphire substrates [67, 69, 72, 102, 189]. These cases demonstrate that the stabilization of metastable phases is not limited to substrates with similar crystal structures but can also be achieved through periodic matching between specific crystallographic planes. These results provide promising opportunities for extending this approach to a broader range of material systems.

Moreover, domain‐matching epitaxy can be used to control the crystallographic orientation of ALD‐grown films. A recent study has demonstrated that, through the orientation engineering of α‐sapphire as a substrate, it was possible to stabilize c‐axis‐oriented rutile TiO_2_, which is typically difficult to achieve [94]. This orientation control led to a significant enhancement in the dielectric performance: while the a‐axis‐oriented films exhibited a dielectric constant of ∼80, the c‐axis‐oriented films achieved values as high as 180.

### 2.3 Grain‐Size Refinement

**FIGURE 4:**
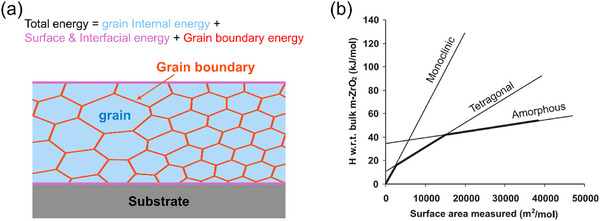
Grain‐size‐induced stabilization of metastable polymorphs. (a) Schematic showing that the total energy of a film is the sum of the internal grain energy, surface energy, and grain boundary energy. (b) Calculated phase stability of ZrO_2_ polymorphs as a function of grain size, indicating a reversal in stability between monoclinic and tetragonal phases at nanoscale dimensions [190]. Reproduced with permission.^[190]^ Copyright 2005, Wiley.

In thin‐film systems, the total thermodynamic energy includes not only the bulk energy of individual grains, but also contributions from the grain boundary energy, surface energy, and interfacial energy with the substrate (Figure 4a). In the absence of kinetic limitations, a phase that minimizes the total energy is theoretically favored. However, as the grain size decreases, the relative contribution of the grain boundary energy becomes more significant, leading to the preferential formation of metastable phases that are not thermodynamically stable in the bulk form.

This phenomenon is particularly pronounced in materials, such as ZrO_2_ and HfO_2_. As shown in Figure 4b, when the grain size of the ZrO_2_ film decreases (i.e., as the interfacial area increases), the total energy of the thermodynamically stable monoclinic phase can become higher than that of the metastable tetragonal phase, resulting in the reversal of their energetic favorability [190]. At even smaller grain sizes, however, crystallization can be hindered and the film may remain amorphous. Indeed, ALD‐grown ZrO_2_ films are observed to form the tetragonal phase over a wide range of growth temperatures and film thicknesses, even without any specific stabilization strategy [174, 175, 176, 177, 178, 179, 180, 181]. However, as the film thickness increases and the grain size grows, these tetragonal films undergo a partial phase transformation into the monoclinic phase [177]. Also, in the case of HfO_2_, the tetragonal phase can be maintained when grain growth is suppressed under specific processing conditions. Table 4 summarizes representative examples of ALD‐grown tetragonal ZrO_2_ and HfO_2_ films, together with the corresponding process windows and film thickness [145, 150, 174, 175, 176, 177, 178, 179, 180, 181].

**TABLE 4:** Representative examples of ALD‐grown tetragonal ZrO_2_ and HfO_2_ films. This table summarizes process windows and film thicknesses where the metastable tetragonal phase is stabilized. In ZrO_2_, the tetragonal phase is easily observed over a broad range of growth temperatures and film thicknesses, whereas in HfO_2_ it is mainly obtained when grain growth is suppressed.

Element	Metastable polymorph	Precursor	Reactant	Growth temperature (°C)	Film thickness (nm)	Refs.
Zr	Fluorite‐ZrO_2_	ZrCl_4_	H_2_O	210‐600	26‐40	[174, 175, 179]
	Fluorite‐ZrO_2_	ZrI_4_	H_2_O_2_	250‐375	42‐125	[176, 178]
	Fluorite‐ZrO_2_	CpZr(NMe_2_)_3_	H_2_O	200‐400	18‐152	[177]
	Fluorite‐ZrO_2_	Zr(NEtMe)_4_	O_3_	225‐300	6‐35	[180, 181]
Hf	Fluorite‐HfO_2_	Hf(NEtMe)_4_	O_3_	200‐220	10	[150]
	Fluorite‐HfO_2_	Hf(NEtMe)_4_	O_2_	280	13	[145]

Compared with ZrO_2_, HfO_2_ requires a much smaller grain size and film thickness to stabilize the tetragonal phase [191]. Consequently, under typical ALD conditions, HfO_2_ tends to crystallize predominantly in the monoclinic phase, whereas a tetragonal phase is rarely observed. However, in HfO_2_ films doped with Ce or La, a tetragonal phase has been reported to form when the grain size is reduced to below ∼4 nm [192]. This suggests that the stabilization of the metastable tetragonal structure is feasible in ALD‐grown HfO_2_ films by precisely controlling the grain size.

The grain size of HfO_2_ films can also be controlled by adjusting the deposition temperature and injection conditions of the oxygen source. For example, in an ALD process using Hf(NEtMe)_4_ and O_3_, grain growth was suppressed at deposition temperatures below 220°C, resulting in the predominant formation of the tetragonal phase [150]. As the temperature increased, grain growth was promoted, inducing a phase transition toward the monoclinic phase. Similarly, when HfO_2_ films were deposited at a relatively high temperature of 280°C with Hf(NEtMe)_4_ and O_2_, the effective suppression of grain growth led to the stable formation of tetragonal HfO_2_ [145]. Accordingly, precise control over grain size is a critical factor in determining the crystal structure of thin films.

### 2.4 Chemical Modulation via Doping and Solid‐Solution Formation

Another effective strategy for achieving phase selectivity is the incorporation of heteroatomic dopants or the formation of solid solutions. Table 5 summarizes representative cases in which metastable phases were stabilized in ALD‐grown films through such chemical modulations [62, 77, 90, 91, 97, 127, 128, 129, 130, 132, 133, 134, 135, 136, 137, 138, 139, 140, 141, 142, 143, 144, 146, 147, 148, 149, 151, 152, 153, 154, 155, 156, 157, 158, 159, 161, 162, 163, 164, 193, 194, 195, 196, 197]. For example, in HfO_2_, the introduction of dopants or solid‐solution formation induces new polymorphic phases. First‐principles calculations have revealed that doping HfO_2_ with various elements (e.g., Si, Ge, Sn, Ti, and Ce) enhances the thermodynamic stability of the tetragonal phase [198, 199]. This effect is primarily attributed to lattice distortion, which becomes more pronounced as the ionic radius of the dopant decreases. Furthermore, doping with trivalent cations introduces oxygen vacancies for charge compensation, further stabilizing the distorted lattice structure. These combined effects lower the free energy of the tetragonal phase, making it more favorable for the formation of the tetragonal phase.

**TABLE 5:** Representative examples of ALD processes for the formation of metastable polymorphs through chemical modulation. This table summarizes cases in which metastable polymorphs are stabilized by doping or forming solid‐solution with specific heteroelements, indicating which heteroelements can be used to access each metastable phase.

Element	Metastable polymorph	Precursor	Heteroelement precursor	Reactant	Growth Temperature (°C)	References
Be	Rocksalt‐BeO	BeMe_2_	Mg(EtCp)_2_	H_2_O	250	[62]
Ti	Rutile‐TiO_2_	Ti(O^i^Pr)_4_	Nb(OEt)_5_	H_2_O	300	[77]
	Rutile‐TiO_2_	Ti(CpMe_5_)(OMe)_3_	SnCl_4_	O_2_ plasma	350	[90]
	Rutile‐TiO_2_	Ti(CpMe_5_)(OMe)_3_	ISN‐02(Sn)	O_3_	350	[91]
Mo	Rutile‐MoO_2_	Unknown	Sn(dmamp)_2_	O_3_	300	[97]
Hf	Fluorite‐HfO_2_	HfCl_4_	AlMe_3_	H_2_O	300	[127]
	Fluorite‐HfO_2_	HfCl_4_	Sr(^t^Bu_3_Cp)_2_	H_2_O	300	[128]
	Fluorite‐HfO_2_	HfCl_4_	Pr(thd)_3_	O_3_	325	[132, 133]
	Fluorite‐HfO_2_	HfCl_4_	Gd(^i^PrCp)_3_	H_2_O	300	[127, 129, 130]
	Fluorite‐HfO_2_	Hf(NMe_2_)_4_	GaMe_3_	O_2_ plasma	270	[135]
	Fluorite‐HfO_2_	Hf(NMe_2_)_4_	Zr(NMe_2_)_4_	H_2_O	250	[134]
	Fluorite‐HfO_2_	Hf(NMe_2_)_4_	Zr(NMe_2_)_4_	O_3_	250‐260	[137, 138]
	Fluorite‐HfO_2_	Hf(NMe_2_)_4_	CpZr(NMe_2_)_3_	O_3_	260‐285	[136, 139]
	Fluorite‐HfO_2_	Hf(NEtMe)_4_	AlMe_3_	H_2_O	200	[140]
	Fluorite‐HfO_2_	Hf(NEtMe)_4_	AlMe_3_	O_2_ plasma	250	[146]
	Fluorite‐HfO_2_	Hf(NEtMe)_4_	AlMe_3_	O_3_	300	[151]
	Fluorite‐HfO_2_	Hf(NEtMe)_4_	V(NEtMe)_4_	H_2_O	240	[141]
	Fluorite‐HfO_2_	Hf(NEtMe)_4_	Si(NMe_2_)_4_	O_2_ plasma	200	[148]
	Fluorite‐HfO_2_	Hf(NEtMe)_4_	SiH(NMe_2_)_3_	H_2_O	200	[140]
	Fluorite‐HfO_2_	Hf(NEtMe)_4_	SiH(NMe_2_)_3_	O_2_ plasma	200	[147]
	Fluorite‐HfO_2_	Hf(NEtMe)_4_	SiH_2_(NEt_2_)_2_	H_2_O	300	[127]
	Fluorite‐HfO_2_	Hf(NEtMe)_4_	Y(MeCp)_3_	O_3_	275‐300	[152, 153]
	Fluorite‐HfO_2_	Hf(NEtMe)_4_	Zr(NEtMe)_4_	H_2_O	250‐300	[142, 143, 144]
	Fluorite‐HfO_2_	Hf(NEtMe)_4_	Zr(NEtMe)_4_	O_3_	260‐280	[143, 154, 155, 156]
	Fluorite‐HfO_2_	Hf(NEtMe)_4_	La(^i^PrCp)_3_	O_2_ plasma	235	[149]
	Fluorite‐HfO_2_	Hf(NEtMe)_4_	La(^i^PrCp)_3_	O_3_	250‐300	[157, 158, 159]
	Fluorite‐HfO_2_	HfCp(NMe_2_)_3_	Y(Cp)(NR_2_)_2_	O_3_	300	[161]
	Fluorite‐HfO_2_	HfCp(NMe_2_)_3_	ZrCp(NMe_2_)_3_	O_3_	300‐320	[162, 163, 164]
	Fluorite‐HfO_2_	Hf(CpMe)_2_(OMe)Me	Y(MeCp)_3_	O_3_	300‐350	[152]

In addition, the tetragonal phase has a relatively low energy barrier for the transition to the orthorhombic phase, which is another metastable phase. Accordingly, ALD‐grown HfO_2_ films often exhibit a mixture of tetragonal and orthorhombic phases [185]. Interestingly, the orthorhombic phase exhibits ferroelectricity owing to its noncentrosymmetric structure. Park et al. experimentally demonstrated the formation of metastable tetragonal and orthorhombic phases in ALD‐grown HfO_2_ films doped with Si, Al, and Gd [127]. Similar observations were reported for doping with other elements, including Al, Si, V, Ga, Sr, Y, La, Pr, and Gd [127, 128, 129, 130, 132, 133, 135, 140, 141, 146, 147, 148, 149, 151, 152, 153, 157, 158, 159, 161].

Owing to the structural similarity between ZrO_2_ and HfO_2_, Hf_x_Zr_1‐x_O_2_ films can form homogeneous solid solutions over a wide compositional range, which facilitates the stabilization of the orthorhombic phase [134, 136, 137, 138, 139, 142, 143, 144, 154, 155, 156, 164]. As the Zr content increases in the Hf_x_Zr_1‐x_O_2_ films, the crystal structure transitions from stable to metastable phases. Notably, ferroelectricity emerges when the Zr concentration reaches approximately 30%–50%, where the transition from monoclinic to orthorhombic occurs [142, 143]. Furthermore, ALD‐grown ZrO_2_/HfO_2_ nanolaminates have been shown to crystallize into the orthorhombic phase, exhibiting ferroelectric behavior [136, 139, 156]. This indicates that nanolaminate structures represent another promising route for stabilizing novel metastable structures.

A strategy has also been proposed to stabilize metastable phases by employing heterostructural materials with the desired crystal structure as a structural framework [62, 77, 90, 91, 97, 162, 163]. Recently, the ALD of thin films incorporating BeO into MgO with a rocksalt structure has been reported to stabilize the highly unstable BeO_6_ octahedron [62]. BeO typically crystallizes into the wurtzite phase with stable BeO_4_ tetrahedra. Its rocksalt phase is highly unstable, with a calculated energy of 0.483 eV atom^−1^, which is much higher than the typical range for experimentally accessible metastable phases [1]. Indeed, rocksalt BeO was not experimentally observed prior to this work. In the reported ALD of Be_x_Mg_1‐x_O solid solutions (x ≤ 0.2), the formation of Be–O octahedra was verified and attributed to the observed enhancement in the dielectric constant.

Similar approaches have been employed to stabilize other metastable phases. For example, the metastable distorted rutile phase of MoO_2_ was stabilized through the formation of a Sn‐Mo‐O solid solution using rutile‐structured SnO_2_ as the structural framework [97]. Additionally, the rutile phase of TiO_2_ was stabilized in ALD‐grown solid‐solution films with rutile SnO_2_ [90, 91] or NbO_2_ [77]. Consequently, these examples highlight the utility of crystallographic templating in which structural similarity enables the stabilization of otherwise inaccessible metastable phases.

## 3 Materials with Different Valence States

The metastable phases are not limited to structural polymorphisms. Compounds composed of the same elements can also exist in different chemical stoichiometries or valence states, and can be considered metastable. Although materials tend to form in their most thermodynamically stable valence states, alternative valence states can emerge during film growth because of various factors. In particular, the reaction paths in ALD can be influenced by the deposition temperature and specific combinations of precursors and reactants, often enabling the stabilization of the metastable valence states of cations in ALD‐grown films. At the atomic scale, valence control in ALD arises because metal‐containing and anion‐containing species are supplied in separate half‐reactions. During the metal‐precursor pulse, chemisorption and ligand elimination generate a partially oxidized, metal‐rich surface intermediate, whereas the subsequent reactant pulse supplies O, S, Se, or N and further oxidizes this intermediate while removing residual ligands. The steady‐state valence of the growing film is therefore governed by the redox balance between these two half‐cycles, which is tuned by the growth temperature, precursor chemistry, and the type and dose of the reactant. This redox‐balance picture is consistent with vanadium‐oxide ALD using V(III) precursors and different oxidants, where adjusting oxidant strength and sequence systematically shifts the film between more reduced VO_x_ compositions and more oxidized V_2_O_5_‐like phases [200].

A wide range of multivalent compounds have been reported using ALD. Table 6 summarizes the representative systems, including oxides (SnO_2_‐SnO, MoO_3_‐MoO_2_, Cu_2_O‐CuO, WO_3_‐W_2_O_3_, Fe_2_O_3_‐Fe_3_O_4_, MnO‐Mn_3_O_4_‐MnO_2_, V_2_O_5_‐VO_2_‐V_2_O_3_, CoO‐Co_3_O_4_, and NiO‐Ni_2_O_3_) [200, 201, 202, 203, 204, 205, 206, 207, 208, 209, 210, 211, 212, 213, 214, 215, 216, 217, 218, 219, 220, 221, 222, 223, 224, 225, 226, 227, 228, 229, 230, 231, 232, 233, 234, 235, 236, 237, 238, 239, 240, 241, 242, 243, 244, 245, 246, 247, 248, 249, 250, 251, 252, 253, 254, 255, 256, 257, 258, 259, 260, 261, 262, 263, 264, 265, 266, 267, 268, 269, 270, 271, 272, 273], chalcogenides (SnS_2_‐SnS, TiS_2_‐TiS_3_, SnSe_2_‐SnSe, CuSe‐Cu_2_Se, and Bi_2_Se_3_‐BiSe) [113, 115, 116, 118, 274, 275, 276, 277, 278, 279, 280, 281, 282, 283, 284, 285, 286, 287, 288, 289, 290, 291, 292, 293, 294, 295, 296, 297, 298, 299, 300, 301, 302, 303, 304, 305], and nitrides (WN‐W_2_N, and TaN‐Ta_3_N_5_) [306, 307, 308, 309, 310, 311, 312, 313, 314, 315, 316, 317]. Additionally, the selective formation of noble metals and their corresponding oxides has been demonstrated using ALD [318, 319, 320, 321, 322, 323, 324, 325, 326, 327, 328, 329, 330, 331, 332, 333, 334, 335, 336, 337, 338, 339, 340, 341, 342, 343, 344, 345, 346]. These behaviors are particularly prominent in transition metal compounds, in which multivalency is inherent.

**TABLE 6:** Overview of ALD processes for controlling valence states in metastable multivalent compounds. This table summarizes ALD processes that link accessible oxidation states to specific precursor/reactant chemistries and process windows, providing a concise guide to which multivalent elements and valence combinations have been demonstrated and where opportunities for unexplored valence states remain.

Element	Multivalent compounds	Precursor	Reactant	Temperature (°C)	References
Ti	TiS_2_	Ti(NMe_2_)_4_	H_2_S	150‐180	[302]
	TiS_2_	Ti(NMe_2_)_4_	H_2_S plasma	100	[303]
	TiS_3_	Ti(NMe_2_)_4_	H_2_S plasma	150‐200	[303]
V	V_2_O_3_	VO(acac)_2_	O_3_	200	[259]
	V_2_O_3_+VO_2_	V(amd)_3_	H_2_O_2_	200	[200]
	V_n_O_2n‐1_	VO(O^i^Pr)_3_	H_2_O	60‐90	[264]
	V_2_O_4_	VO(O^n^Pr)_3_	CH_3_COOH	200	[266]
	VO_2_	V(NEtMe)_4_	O_3_	150	[260, 261, 262, 263, 265]
	VO_2_	V(amd)_3_	H_2_O_2_+H_2_	200	[200]
	VO_2_	V(NEtMe)_4_	H_2_O	150	[262, 265]
	VO_2_	VO(O^i^Pr)_3_	H_2_O	60‐135	[264, 267]
	V_6_O_13_	V(NEtMe)_4_	O_3_	150	[261, 262]
	V_4_O_9_	V(NEtMe)_4_	H_2_O	150	[262]
	V_3_O_7_	V(NEtMe)_4_	O_3_	150	[262]
	V_2_O_5_	VO(acac)_2_	O_3_	200	[259]
	V_2_O_5_	V(amd)_3_	O_3_	200	[200]
	V_2_O_5_	V(amd)_3_	H_2_O+O_2_	200	[200]
	V_2_O_5_	V(amd)_3_	H_2_O_2_	200	[200]
	V_2_O_5_	V(NEtMe)_4_	O_3_	150	[265]
	V_2_O_5_	V(NEtMe)_4_	H_2_O	150	[265]
	V_2_O_5_	V(NEtMe)_4_	O_3_+H_2_O	150	[262]
	V_2_O_5_	VO(O^i^Pr)_3_	H_2_O	135	[267]
	VS	V(NEtMe)_4_	H_2_S	200‐300	[305]
	V_2_S_3_	V(amd)_3_	H_2_S	150‐200	[304]
	VS_4_	V(amd)_3_	H_2_S	225‐250	[304]
Cr	Cr_2_O_3_	Cr(acac)_3_	O_3_	300‐350	[210]
	Cr_2_O_3_+CrO_3_	CrO_2_Cl_2_	H_2_O	100‐200	[211, 212]
	Cr_2_O_3_+CrO_3_	CrO_2_Cl_2_	H_2_O_2_	150‐200	[211, 212]
	Cr_2_O_3_+CrO_3_	CrO_2_Cl_2_	H_2_O+H_2_O_2_	150	[211]
	Cr_2_O_3_+CrO_3_	CrO_2_Cl_2_	CH_3_OH	150	[211]
Mn	MnO	Mn(EtCp)_2_	H_2_O	100‐300	[232]
	MnO	Mn(thd)_3_	NH_3_ plasma	180	[235]
	Mn_3_O_4_	Mn(^t^BuAMD)_2_	H_2_O	150	[234]
	Mn_3_O_4_	Mn(thd)_3_	H_2_O	180	[235]
	Mn_3_O_4_	Mn(thd)_3_	O_3_	≥240	[236]
	Mn_3_O_4_	Mn_2_(CO)_10_	O_3_	120‐160	[237]
	Mn_3_O_4_+MnO	Mn(thd)_3_	H_2_ plasma	180	[235]
	Mn_2_O_3_	Mn(^t^BuAMD)_2_	H_2_O	175‐250	[234]
	Mn_2_O_3_+MnO	Mn_2_(CO)_10_	O_3_	60‐100	[237]
	Mn_5_O_8_	Mn(EtCp)_2_	O_3_	150‐180	[233]
	MnO_2_	Mn(thd)_3_	O_3_	140‐230	[235, 236]
Fe	Fe	Fe(^t^BuAMD)_2_	H_2_	250	[203]
	Fe	FeCp(Cp^t^Bu)	O_2_ plasma	150‐250	[230]
	FeO	Fe(^t^BuAMD)_2_	H_2_O	250	[203]
	Fe_3_O_4_	Fe(2,4‐C_7_H_11_)_2_	H_2_O_2_	120	[226]
	Fe_3_O_4_	Fe_2_(O^t^Bu)_6_	H_2_O	130‐170	[229]
	Fe_3_O_4_	FeCp(Cp^t^Bu)	O_2_ plasma	150‐250	[230]
	Fe_3_O_4_	FeCp_2_	O_2_	400	[231]
	Fe_3_O_4_+Fe_2_O_3_	FeCp_2_	O_2_	350‐500	[227]
	Fe_2_O_3_	Fe(2,4‐C_7_H_11_)_2_	O_3_	120	[226]
	Fe_2_O_3_	FeCp_2_	O_2_	>500	[227]
	Fe_2_O_3_	FeCp_2_	O_3_	200	[228]
	Fe_2_O_3_	Fe_2_(O^t^Bu)_6_	H_2_O	130‐170	[229]
	Fe_2_O_3_	FeCp(Cp^t^Bu)	O_2_ plasma	150‐250	[230]
	Fe_2_O_3_	FeCp_2_	O_2_	400	[231]
	FeS	Fe(amd)_2_	H_2_S	80‐250	[287]
	FeS_2_	Fe(amd)_2_	H_2_S plasma	80‐200	[286]
	FeSe	Fe(^i^PrAMD)_2_	Se(SiEt_3_)_2_	350‐370	[288]
	Fe_3_Se_4_	Fe(^i^PrAMD)_2_	Se(SiEt_3_)_2_	390‐450	[288]
Co	Co	Co(^i^PrAMD)_2_	H_2_	350	[203]
	Co	Co(AMD)_2_	H_2_O	180‐270	[206]
	Co	CoCl_2_(TMEDA)	H_2_O	225‐300	[208]
	Co_3_O_4_	Co(^i^PrAMD)_2_	O_3_	225‐250	[204]
	Co_3_O_4_	Co_2_(CO)_8_	O_3_	50	[205]
	Co_3_O_4_	CoCp_2_	O_3_	150‐280	[209]
	Co_3_O_4_+CoO	Co_2_(CO)_8_	O_3_	50	[205]
	Co_3_O_4_+CoO	CoCp_2_	O_3_	331	[209]
Cu	Cu	[Cu(^s^Bu‐amd)]_2_	H_2_ plasma	150	[217]
	Cu	(hfac)Cu(I)(DMB)	H_2_ plasma+O_2_ plasma	100	[220]
	Cu	Cu(I)(hfac)(tmvs)	N_2_	160	[224]
	Cu_2_O	Cu(P^n^Bu_3_)_2_(acac)	H_2_O + O_2_	110‐125	[215]
	Cu_2_O	Cu(dmap)_2_	O_3_	80‐140	[216]
	Cu_2_O	[Cu(^s^Bu‐amd)]_2_	H_2_ plasma+O_2_ plasma	150	[217]
	Cu_2_O	[Cu(^s^Bu‐amd)]_2_	H_2_O	180	[218]
	Cu_2_O	Cu(dmamb)_2_	H_2_O	140‐160	[219]
	Cu_2_O	(hfac)Cu(I)(DMB)	H_2_ plasma+O_2_ plasma	100	[220]
	Cu_2_O	Cu(dmap)_2_	H_2_O	110‐175	[221]
	Cu_2_O	Cu(hfac)_2_	H_2_O	280	[222]
	Cu_2_O	Cu(OAc)_2_	H_2_O	180‐220	[223]
	Cu_2_O	Cu(I)(hfac)(tmvs)	H_2_O	220	[224]
	Cu_2_O	Cu(acac)_2_	O_2_+H_2_O	200	[225]
	Cu_2_O+CuO	(hfac)Cu(I)(DMB)	O_3_	100	[214]
	CuO	Cu(acac)_2_	O_3_	150‐230	[213]
	CuO	Cu(dmap)_2_	O_3_	80‐140	[216]
	CuO	[Cu(^s^Bu‐amd)]_2_	O_2_ plasma	150	[217]
	CuO	(hfac)Cu(I)(DMB)	H_2_ plasma+O_2_ plasma	100	[220]
	CuO	Cu(I)(hfac)(tmvs)	O_3_	260	[224]
	Cu_2_Se	Cu(II) pivalate	(Et_3_Si)_2_Se	300	[275]
	Cu_2_Se	CuCl	(Et_3_Si)_2_Se	400	[275]
	CuSe	Cu(II) pivalate	(Et_3_Si)_2_Se	165	[275]
	Cu_2_S	[Cu(^s^Bu‐amd)]_2_	H_2_S	130‐150	[276, 277, 278]
	Cu_2_S	CuAMD	H_2_S	135	[279]
	Cu_2_S	Cu(acac)_2_	H_2_S	130‐200	[280]
	Cu_2_S	Cu(dmamb)_2_	H_2_S	120‐200	[282]
	Cu_1.8_S	Cu(thd)_2_	H_2_S	175‐280	[284, 285]
	CuS	Cu(acac)_2_	Solid S	140‐160	[281]
	CuS	Cu(thd)_2_	H_2_S	125‐175	[283, 284, 285]
Nb	NbO_2_	Nb‐TBTEA	H_2_O	200	[241]
	NbO_2_	Nb(OEt)_5_	H_2_O	170‐300	[243]
	NbO_2_+Nb_2_O_5_	Nb(OEt)_5_	H_2_O	170‐300	[243]
	Nb_2_O_5_	Nb‐TBTEA	H_2_O	200	[241]
	Nb_2_O_5_	^t^BuN=Nb(NEt_2_)_3_	H_2_O+O_3_	150‐325	[242]
	Nb_2_O_5_	^t^BuN=Nb(NMeEt)_3_	H_2_O+O_3_	150‐325	[242]
	Nb_2_O_5_	Nb(OEt)_5_	H_2_O	170‐300	[243]
Mo	MoO_2_	MoO_2_(thd)_2_	O_3_	225	[238]
	MoO_2_	Mo(NMe_2_)_4_	O_2_	150	[239]
	MoO_x_ (2<x<3)	Mo(NMe_2_)_4_	H_2_O+O_3_	80	[240]
	MoO_3_	MoO_2_(thd)_2_	O_3_	225	[238]
	MoO_3_	Mo(NMe_2_)_4_	O_3_	150	[239]
Sn	SnO	Sn(dmamb)_2_	H_2_O	100‐210	[244, 245]
	SnO	Sn(dmamp)_2_	H_2_O	150‐210	[246, 247, 248, 258]
	SnO	Sn(edpa)_2_	H_2_O	70‐300	[249]
	SnO	Sn(^i^Pr_2_fAMD)_2_	H_2_O	220‐240	[250]
	SnO	Sn(N(SiMe_3_)_2_)_2_	H_2_O	100‐250	[251]
	SnO	Sn(TAA)_2_	H_2_O	100‐180	[252]
	SnO_2_	Sn(edpa)_2_	O_2_ plasma	70‐300	[249]
	SnO_2_	Sn(N(SiMe_3_)_2_)_2_	O_3_	80‐200	[251]
	SnO_2_	Sn(NMe_2_)_4_	H_2_O	30‐300	[253, 254]
	SnO_2_	SnCl_4_	H_2_O	300‐600	[255, 256, 257]
	SnO_2_	Sn(dmamp)_2_	H_2_O_2_	100‐200	[258]
	SnS	Sn(NMe_2_)_4_	H_2_S	100‐240	[292, 295, 297]
	SnS	Sn(η^2^‐((NBu^t^)CHMeCHMe(NBu^t^)))_2_	H_2_S	50‐125	[293]
	SnS	Sn(η^2^‐MeC(NPr^i^)_2_)_2_	H_2_S	100‐250	[293]
	SnS	Sn(dmamp)_2_	H_2_S plasma	210	[298]
	SnS_2_	Sn(NMe_2_)_4_	H_2_S	140‐180	[292, 295]
	SnS_2_	Sn(dmamp)_2_	H_2_S plasma	150‐240	[294]
	SnSe	Sn(NMe_2_)_4_	Se(SiMe_3_)_2_	170‐190	[299]
	SnSe	SnCl_4_	Sn(SiEt_3_)_2_	180‐200	[300]
	SnSe	SnEt_4_	H_2_Se	250‐400	[301]
	SnSe_2_	Sn(NMe_2_)_4_	Se(SiMe_3_)_2_	110‐150	[299]
W	W_2_O_3_	W_2_(NMe_2_)_6_	H_2_O	160‐200	[268]
	WO_3_	WCO_6_	H_2_O	300	[269, 270]
	WO_3_	W(N^t^Bu)_2_(NMe_2_)_2_	H_2_O	250‐350	[271, 272]
	WO_3_	WO_2_(^t^BuAMD)_2_	H_2_O	150‐270	[273]
	W_2_N	W(CO)_6_	NH_3_	180‐195	[314]
	W_x_N (1.1≤x≤1.4)	WF_6_+B_2_H_6_	NH_3_	300	[315]
	WN	WCl_5_	N_2_/H_2_ plasma	250	[317]
	WN_1.56_	W(CO)(3‐hexyne)_3_	N_2_/NH_3_ plasma	250	[313]
	WN	WCl_5_	N_2_/H_2_ plasma	250	[317]
	W_2_N	WCl_5_	N_2_/H_2_ plasma	250	[317]
	WN_3.32_	W(N^t^Bu)_2_(NMe_2_)_2_	N_2_ plasma	250	[316]
	W_x_N (1.13≤x≤1.93)	W(N^t^Bu)_2_(NMe_2_)_2_	NH_3_ plasma	174‐400	[316]
	W_x_N (1.26≤x≤3.19)	W(N^t^Bu)_2_(NMe_2_)_2_	N_2_/H_2_ plasma	250	[316]
	W_x_N (1.9≤x≤3.76)	W(N^t^Bu)_2_(NMe_2_)_2_	N_2_/H_2_ plasma	250‐400	[316]
Ta	Ta_2_N	Ta(NMe_2_)_5_	H_2_ plasma	225	[307]
	TaN	Ta(N^i^Pr)(NEtMe)_3_	N_2_/H_2_ plasma	400	[306]
	TaN	TaCl_5_	NH_3_+Zn	400‐500	[309]
	TaN	TaF_5_	H_2_ plasma+NH_3_	<250	[311]
	TaN	TaF_5_	H_2_ plasma+NH_3_+H_2_	200	[312]
	Ta_2_N_3_	Ta(NMe_2_)_5_	N_2_/H_2_ plasma	225	[307]
	Ta_3_N_5_	Ta(N^i^Pr)(NEtMe)_3_	N_2_/H_2_ plasma	400	[306]
	Ta_3_N_5_	TaCl_5_	NH_3_	200‐500	[309]
	Ta_3_N_5_	TaF_5_	H_2_ plasma+NH_3_	300‐350	[311]
	Ta_3_N_5_	TaF_5_	H_2_ plasma+NH_3_/H_2_ plasma	200	[312]
	TaN_x_ (0.88≤x≤1.35)	TaCp(=N^t^Bu)(NEt_2_)_2_	N_2_/H_2_ plasma	390‐450	[308]
	TaN_x_ (1≤x≤1.7)	TaF_5_	NH_3_	350	[310]
	TaN_x_ (1.53≤x≤1.79)	Ta(=N^t^Bu)(NEt_2_)_3_	NH_3_	240‐280	[308]
Bi	BiSe	Bi(NMe_2_)_3_	Se(SnMe_3_)_2_	90‐120	[274]
	BiSe_2_	Bi(NMe_2_)_3_	Se(SnMe_3_)_2_	150	[274]

The ability to stabilize different valence states within a single‐material system offers significant opportunities for tuning electrical, optical, and chemical properties, thereby increasing the flexibility of material design. A representative case is tin oxide: while SnO_2_, the thermodynamically stable phase, behaves as an n‐type semiconductor, the metastable SnO exhibits p‐type conductivity [244, 245, 246, 247, 248, 249, 250, 251, 252, 253, 254, 255, 256, 257]. This polarity contrast in the same material system offers new possibilities of novel electronic devices, including p–n junctions and complementary electronics.

In ALD, several approaches have been employed to selectively control metal valence states, including variations in the deposition temperature, the choice of precursors and reactants, and post‐deposition treatments. In this section, we highlight these strategies and examine representative examples of valence‐controlled metastable‐phase formation via ALD.

### 3.1 Control of Deposition Temperature

The deposition temperature is a critical factor that determines the valence states of metal cations in ALD‐grown films. Changes in the deposition temperature can alter the precursor reactivity, decomposition behavior, oxidation potential, and volatility of specific elements, potentially resulting in variations in the film composition. Table 7 summarizes the representative systems in which the valence states of the metal ions in the ALD‐grown films varied with the deposition temperature [201, 202, 209, 212, 227, 234, 236, 237, 274, 275, 284, 285, 288, 292, 295, 299, 303, 304, 308, 311, 316]. In certain cases, an increase in the deposition temperature can lead to a higher valence state of the metal ions in the films. For instance, in ALD using FeCp_2_ and O_2_, Fe_2_O_3_ and metastable Fe_3_O_4_ comprising Fe^2+^ and Fe^3+^ ions were formed at relatively low temperatures (350–500°C), whereas only Fe_2_O_3_ was formed at temperatures above 500°C [227]. A similar trend was observed for the ALD of Bi–Se compounds using Bi(NMe_2_)_3_ and Se(SnMe_3_)_2_. The metastable BiSe phase was stabilized in the temperature range of 90–120°C, whereas the stable Bi_2_Se_3_ phase was observed at elevated temperatures. [274] In another example, using Fe(^i^PrAMD)_2_ and Se(SiEt_3_)_2_, FeSe was formed at 350°C. However, when the deposition temperature exceeded 370°C, the thermal decomposition of Fe(^i^PrAMD)_2_ reduced the supply of Fe ions, leading to the formation of metastable Fe_3_Se_4_ [288].

**TABLE 7:** Representative examples of ALD processes for controlling valence states in metastable multivalent compounds via the modulation of deposition temperature. This table summarizes how arranging oxidation states as a function of growth temperature makes it clear which thermal regimes favor more reduced or more oxidized valence states in each material system.

Element	Material	Precursor	Reactant	Temperature (°C)	References
Ti	TiS_3_	Ti(NMe_2_)_4_	H_2_S plasma	100	[303]
	TiS_2_	Ti(NMe_2_)_4_	H_2_S plasma	150‐200	[303]
V	VS_4_	V(amd)_3_	H_2_S	150‐200	[304]
	V_2_S_3_	V(amd)_3_	H_2_S	225‐250	[304]
Cr	CrO_x_ (Cr(III)/Cr(VI) = 0.5‐0.7)	CrO_2_Cl_2_	H_2_O	100	[212]
	CrO_x_ (Cr(III)/Cr(VI) = 2)	CrO_2_Cl_2_	H_2_O	150	[212]
	CrO_x_ (Cr(III)/Cr(VI) = 1.2‐1.3)	CrO_2_Cl_2_	H_2_O+H_2_O_2_	150	[212]
	CrO_x_ (Cr(III)/Cr(VI) = 10‐12)	CrO_2_Cl_2_	H_2_O+H_2_O_2_	200	[212]
Mn	Mn_3_O_4_	Mn(^t^BuAMD)_2_	H_2_O	150	[234]
	Mn_2_O_3_+MnO	Mn(^t^BuAMD)_2_	H_2_O	175‐250	[234]
	MnO_2_	Mn(thd)_3_	O_3_	140‐230	[236]
	Mn_3_O_4_	Mn(thd)_3_	O_3_	≥240	[236]
	Mn_2_O_3_	Mn_2_(CO)_10_	O_3_	60‐100	[237]
	Mn_3_O_4_	Mn_2_(CO)_10_	O_3_	120‐160	[237]
Fe	Fe_2_O_3_+Fe_3_O_4_	FeCp_2_	O_2_	350‐500	[227]
	Fe_2_O_3_	FeCp_2_	O_2_	>500	[227]
	FeSe	Fe(^i^PrAMD)_2_	Se(SiEt_3_)_2_	350‐370	[288]
	Fe_3_Se_4_	Fe(^i^PrAMD)_2_	Se(SiEt_3_)_2_	390‐450	[288]
Co	Co_3_O_4_	CCTBA	O_3_	68	[201]
	Co_3_O_4_+CoO	CCTBA	O_3_	80‐138	[201]
	Co_3_O_4_+CoO	Co(^i^Pr_2_DAD)_2_	O_2_	125‐250	[202]
	Co_3_O_4_	Co(^i^Pr_2_DAD)_2_	O_2_	≥265	[202]
	Co_3_O_4_	CoCp_2_	O_3_	150‐280	[209]
	Co_3_O_4_+CoO	CoCp_2_	O_3_	331	[209]
Cu	CuSe	Cu(II) pivalate	(Et_3_Si)_2_Se	165	[275]
	Cu_2_Se	Cu(II) pivalate	(Et_3_Si)_2_Se	300	[275]
	CuS	Cu(thd)_2_	H_2_S	125‐175	[284, 285]
	Cu_1.8_S	Cu(thd)_2_	H_2_S	175‐280	[284, 285]
Sn	SnS_2_	TDMASn	H_2_S	140‐150	[292]
	SnS	TDMASn	H_2_S	160‐180	[292]
	SnS_2_	TDMASn	H_2_S	180	[295]
	SnS	TDMASn	H_2_S	240	[295]
	SnSe_2_	Sn(NMe_2_)_4_	Se(SiMe_3_)_2_	110‐150	[299]
	SnSe	Sn(NMe_2_)_4_	Se(SiMe_3_)_2_	170‐190	[299]
W	WN	W(N^t^Bu)_2_(NMe_2_)_2_	NH_3_ plasma	175	[316]
	W_2_N	W(N^t^Bu)_2_(NMe_2_)_2_	NH_3_ plasma	400	[316]
	W_2_N	W(N^t^Bu)_2_(NMe_2_)_2_	N_2_/H_2_ plasma	250	[316]
	W_3.76_N	W(N^t^Bu)_2_(NMe_2_)_2_	N_2_/H_2_ plasma	400	[316]
Ta	TaN_0.88_	TaCp(=N^t^Bu)(NEt_2_)_2_	NH_3_	390	[308]
	TaN_1.35_	TaCp(=N^t^Bu)(NEt_2_)_2_	NH_3_	450	[308]
	TaN_1.53_	Ta(=N^t^Bu)(NEt_2_)_3_	NH_3_	240	[308]
	TaN_1.79_	Ta(=N^t^Bu)(NEt_2_)_3_	NH_3_	280	[308]
	TaN	TaF_5_	H_2_ plasma+NH_3_	<250	[311]
	Ta_3_N_5_	TaF_5_	H_2_ plasma+NH_3_	300‐350	[311]
Bi	BiSe	Bi(NMe_2_)_3_	Se(SnMe_3_)_2_	90‐120	[274]
	BiSe_2_	Bi(NMe_2_)_3_	Se(SnMe_3_)_2_	150	[274]

In contrast, the valence states of the metal cations in ALD‐grown films decrease with increasing deposition temperature. In the ALD process using Mn_2_(CO)_10_ and O_3_, Mn_2_O_3_ was formed at low temperatures (60–100°C), whereas Mn_3_O_4_, which contains both Mn^2+^ and Mn^3+^, was formed at high temperatures (120–160°C) [237]. Similarly, in the ALD of cobalt oxides using CoCp_2_ and O_3_, stoichiometric Co_3_O_4_ (with Co^2+^ and Co^3+^) was formed at 150–285°C. However, at 331°C, incomplete oxidation owing to the decomposition of CoCp_2_ increased the proportion of Co^2+^, leading to a mixture of Co_3_O_4_ and metastable CoO [209]. This tendency is particularly notable in materials containing volatile anions, such as S or Se, where higher temperatures can induce anion desorption and promote a reduction in the valence states of metal cations. For instance, in ALD using Sn(N(CH_3_)_2_)_4_ and Se(Si(CH_3_)_3_)_2_, the stable SnSe_2_ phase was formed at 110–150°C, whereas the high‐temperature phase SnSe emerged above 170°C [299]. In ALD using V(amd)_3_ and H_2_S, metastable VS_4_ was obtained at 150–200°C owing to the oxidation of V^3+^ by disulfide (S_2_^2−^) species generated from reactions between H_2_S and –SH ligands [304]. However, at higher temperatures (225–250°C), the thermal decomposition of S_2_^2−^ inhibited this reaction path, and the surface reaction proceeded via only H_2_S, leading to the formation of stable V_2_S_3_ [304].

Changes in the deposition temperature can also alter the chemical nature of the reactants themselves [236, 303]. This effect was demonstrated in the ALD of metastable TiS_3_ containing S_2_^2−^ [303]. In ALD using Ti(NMe_2_)_4_ and H_2_S plasma, TiS_3_ was synthesized at 100°C. However, the disulfide species decomposed at 150–200°C, thereby yielding TiS_2_ [303]. A similar temperature‐driven transition was observed in the ALD of manganese oxides using Mn(thd)_3_ and O_3_ [236]. The MnO_2_ phase with Mn^4+^ was formed at the low temperatures of 140–230°C, whereas above 240°C, the reduced lifetime of O_3_ diminished its oxidizing power, leading to the formation of metastable Mn_3_O_4_, which contains both Mn^2+^ and Mn^3+^ [236].

### 3.2 Precursor‐Based Control of the Valence State of Films

In ALD reactions based on ligand‐exchange mechanisms, the valence state of the metal center in the precursor is preserved in the resulting film. This occurs when the ligands in the precursor are removed without combustion, thereby preventing further oxidation of the metal ions. This approach has enabled the synthesis of metastable phases of compounds containing multivalent transition metals, such as V, Mn, Co, Cu, Sn, and W (Table 8) [113, 115, 116, 118, 208, 216, 219, 220, 233, 245, 246, 247, 248, 249, 253, 254, 255, 256, 257, 258, 261, 262, 269, 277, 280, 282, 284, 290, 291, 292, 293, 294].

**TABLE 8:** Representative examples of ALD processes for controlling valence states in metastable multivalent compounds by tailoring the valence state of the metal in the precursor. This table summarizes how choosing precursors with different formal oxidation states or ligand environments systematically biases the resulting films toward particular valence states, providing practical design rules for precursor selection when targeting metastable oxidation states.

Element	Material	Metal oxidation state in precursor	Precursor	Reactant	Temperature (°C)	References
V	VO_2_	4+	V(NEtMe)_4_	O_3_	150	[260, 261]
Mn	MnO	2+	Mn(EtCp)_2_	H_2_O	100‐300	[232]
Co	CoO	2+	Co(BTSA)_2_(THF)	H_2_O	75‐250	[207]
Cu	Cu_2_O	1+	Cu(P^n^Bu_3_)_2_(acac)	H_2_O + O_2_	110‐125	[215]
	Cu_2_O	1+	[Cu(^s^Bu‐amd)]_2_	H_2_O	180	[218]
	Cu_2_O	1+	Cu(dmamb)_2_	H_2_O	140‐160	[219]
	Cu_2_S	1+	[Cu(^s^Bu‐amd)]_2_	H_2_S	130‐150	[276, 277]
	Cu_2_S	1+	Cu_2_DBA	H_2_S	135	[278]
	Cu_2_S	1+	CuAMD	H_2_S	135	[279]
	CuS	2+	Cu(acac)_2_	Solid S	140‐160	[281]
	CuS	2+	Cu(thd)_2_	H_2_S	125‐160	[283]
Sn	SnO	2+	Sn(dmamb)_2_	H_2_O	100‐210	[244, 245]
	SnO	2+	Sn(dmamp)_2_	H_2_O	150‐210	[246, 247, 248]
	SnO	2+	Sn(^i^Pr_2_fAMD)_2_	H_2_O	220‐240	[250]
	SnO	2+	Sn(TAA)_2_	H_2_O	100‐210	[252]
	SnO_2_	4+	TDMASn	H_2_O	30‐300	[253, 254]
	SnO_2_	4+	SnCl_4_	H_2_O	300‐600	[255, 256, 257]
	SnS	2+	Sn(acac)_2_	H_2_S	80‐175	[116, 289]
	SnS	2+	Sn(dmamp)_2_	H_2_S	90‐285	[115, 290]
	SnS	2+	Sn(dmpa)_2_	H_2_S	25‐250	[118]
	SnS	2+	Sn(amd)_2_	H_2_S	100‐200	[291]
	SnS	2+	Sn(^i^Pr_2_fAMD)_2_	H_2_S	65‐200	[113]
	SnS	2+	Sn(η^2^ ‐((NBu^t^)CHMeCHMe(NBu^t^)))_2_	H_2_S	50‐125	[293]
	SnS	2+	Sn(η^2^ ‐MeC(NPr^i^)_2_)_2_	H_2_S	100‐250	[293]
W	W_2_O_3_	3+	W_2_(NMe_2_)_6_	H_2_O	160‐200	[268]

Among these systems, tin oxides are representative materials in which the resulting phase—either SnO or SnO_2—_is determined by the valence state of the metal in the precursor. Various precursors that contain either Sn^2+^ or Sn^4+^ ions are available [244, 245, 246, 247, 248, 252, 253, 254, 255, 256, 257]. Precursors containing Sn^4+^, such as SnCl_4_, SnI_4_, and TDMASn, react with H_2_O to form the stable SnO_2_ [253, 254, 255, 256, 257]. In contrast, precursors containing Sn^2+^, including Sn(dmamp)_2_, Sn(dmamb)_2_, Sn(edpa)_2_, and Sn(OtAmyl)_2_, react with H_2_O to yield the metastable SnO [244, 245, 246, 247, 248, 252]. A similar trend was observed in the formation of tin sulfide films [113, 115, 116, 118, 289, 290, 291, 292, 293]. The ALD with a Sn^4+^ precursor of TDMASn and H_2_S leads to the formation of n‐type SnS_2_ at 60–150°C [292], whereas the use of Sn^2+^ precursors, such as Sn(η^2^‐((NtBu)CHMeCHMe(NtBu)))_2_, Sn(dmamp)_2_, Sn(dmpa)_2_, Sn(^i^PrAMD)_2_, and Sn(^i^PrFMD)_2_, yields metastable p‐type SnS under similar conditions [113, 115, 116, 118, 289, 290, 291, 292, 293].

However, this precursor‐based strategy for controlling valence states may become ineffective at elevated temperatures. At high temperatures, the partial thermal decomposition of the precursor or enhanced reactivity with the co‐reactant may lead to unintended oxidation. For example, in ALD using Sn(dmamb)_2_ with Sn^2+^, SnO was formed at 100–150°C, but SnO_2_ also appeared in the films deposited at 200°C [244].

Conversely, a certain ALD process intentionally exploit the thermal instability of a precursor. In such case, a thermodynamically unstable precursor self‐decomposes via a disproportionation reaction in an inert atmosphere, allowing metal deposition without a co‐reactant [224]. A notable example is the deposition of metallic Cu using the low‐stability precursor Cu(I)(hfac)(tmvs). Above 60°C, it undergoes the following disproportionation reaction:

(1) $$2\mathrm{Cu}\left(I\right)\left(\mathrm{hfac}\right)\;\left(\mathrm{tmvs}\right)=\mathrm{Cu}\left(0\right)+\mathrm{Cu}\left(\mathrm{II}\right){\left(\mathrm{hfac}\right)}_{2}+2(\mathrm{tmvs})$$

Through this reaction, the byproducts of Cu(II)(hfac)_2_ and tmvs are volatilized at 160°C under a N_2_ atmosphere, enabling the deposition of high‐purity Cu metal [224].

### 3.3 Reactant‐Based Control of the Valence State of Films

**FIGURE 5:**
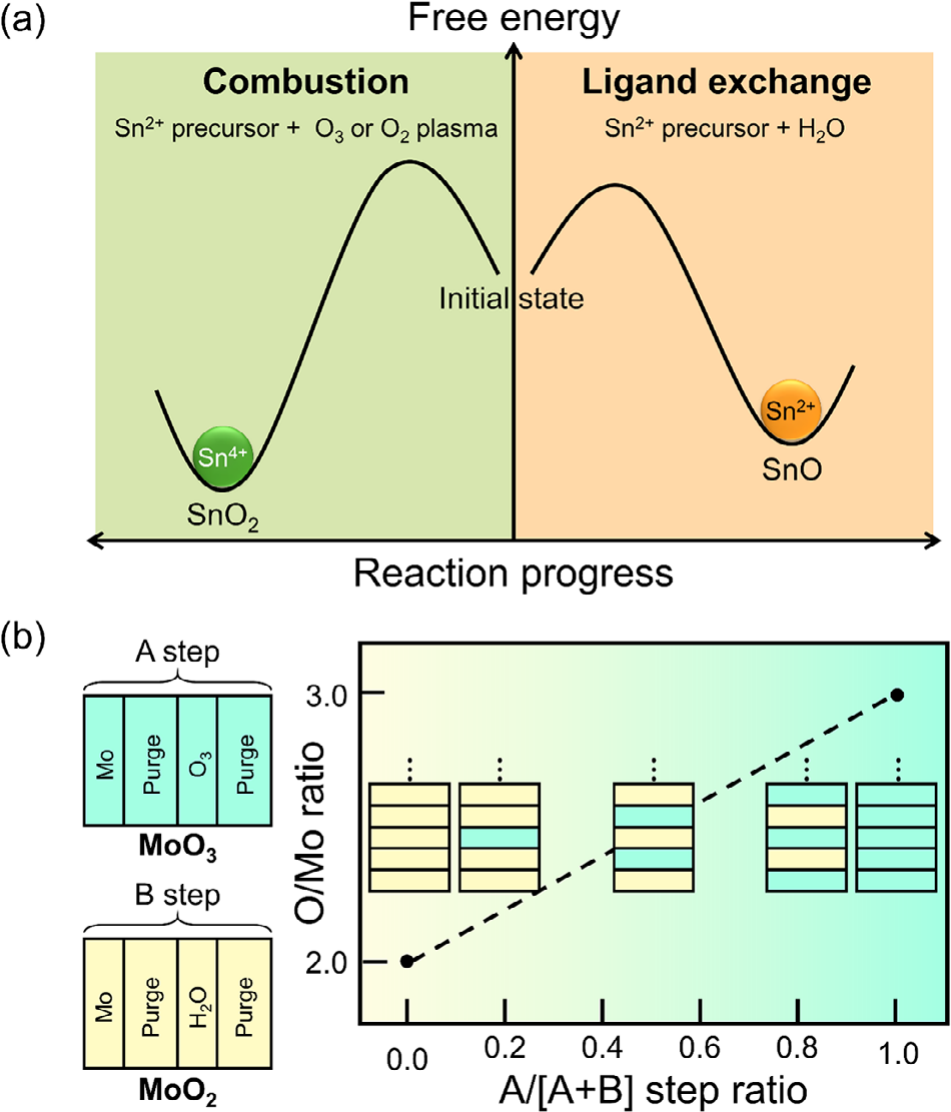
Reactant‐based strategies for controlling valence states in ALD. (a) Schematic of valence state evolution in the ALD of Sn‐based compounds using precursors with Sn^2+^, depending on the type of reactant. (b) Strategy for modulating the valence states of Mo ions in MoO_x_ via alternating ALD cycles using H_2_O and O_3._

The valence state of metal ions in ALD‐grown films can also vary depending on the choice of the co‐reactant. Table 9 summarizes representative ALD processes in which these effects were observed [115, 203, 204, 211, 217, 220, 224, 226, 231, 235, 240, 249, 251, 258, 286, 287, 290, 294, 307, 309, 310, 312, 316, 317]. Such differences are likely attributed to the adoption of different reaction pathways depending on the choice of the co‐reactant. For example, as discussed above, ALD reactions using Sn precursors containing Sn^2+^ with H_2_O or H_2_S as co‐reactants typically yield SnO or SnS films via the ligand‐exchange mechanism [113, 115, 116, 118, 244, 245, 246, 247, 248, 252, 289, 290, 291, 293]. However, when other reactants, such as O_2_ plasma, O_3_, or H_2_S plasma, are used with the same Sn^2+^ precursors, SnO_2_ or SnS_2_ films are formed owing to the increase in the valence state of Sn ions (Figure 5a) [249, 251, 294]. Furthermore, the valence states of metal ions in ALD‐grown films can vary, even with changes in the reactant dose supplied during each ALD cycle. For example, in the ALD of iron oxides using Fe(Cp)_2_ and O_2_ at 400°C, increasing the O_2_ pulse time from 1 s to 4 s induced a phase transition from Fe_3_O_4_ to Fe_2_O_3_, reflecting an increase in the valence state of Fe ions [231]. In addition, in the ALD of TaN_x_ using TaF_5_ and H_2_/N_2_ plasma, the valence state of Ta ions in the films has been reported to vary with the N_2_/(H_2_+N_2_) ratio in the reactant plasma [311].

**TABLE 9:** Representative examples of ALD processes for controlling valence states in metastable multivalent compounds through the selection of the reactants. This table summarizes how the choice of oxidizing, reducing, or complex‐forming reactants, together with their dose conditions, shifts the balance between competing valence states in the resulting films.

Element	Material	Precursor	Reactant	Temperature (°C)	Refs.
Cr	CrO_x_ (Cr(III)/Cr(VI)>10)	CrO_2_Cl_2_	CH_3_OH	150	[211]
	CrO_x_ (Cr(III)/Cr(VI)=2)	CrO_2_Cl_2_	H_2_O	150	[211]
	CrO_x_ (Cr(III)/Cr(VI)=1.2‐1.3)	CrO_2_Cl_2_	H_2_O_2_	150	[211]
Mn	MnO	Mn(thd)_3_	NH_3_ plasma	180	[235]
	Mn_3_O_4_+MnO	Mn(thd)_3_	H_2_ plasma	180	[235]
	Mn_3_O_4_	Mn(thd)_3_	H_2_O	180	[235]
	MnO_2_	Mn(thd)_3_	O_3_	180	[235]
Fe	Fe_3_O_4_	Fe(2,4‐C_7_H_11_)_2_	H_2_O_2_	120	[226]
	Fe_2_O_3_	Fe(2,4‐C_7_H_11_)_2_	O_3_	120	[226]
	Fe	Fe(^t^BuAMD)_2_	H_2_	250	[203]
	FeO	Fe(^t^BuAMD)_2_	H_2_O	250	[203]
	Fe_3_O_4_	FeCp_2_	O_2_ (1s pulse)	400	[231]
	Fe_2_O_3_	FeCp_2_	O_2_ (4s pulse)	400	[231]
	FeS_2_	Fe(amd)_2_	H_2_S plasma	80‐200	[286]
	FeS	Fe(amd)_2_	H_2_S	80‐250	[287]
Co	Co	Co(^i^PrAMD)_2_	H_2_	350	[203]
	CoO	Co(^i^PrAMD)_2_	O_2_	250	[203]
	CoO	Co(^i^PrAMD)_2_	H_2_O	150‐250	[204]
	Co_3_O_4_	Co(^i^PrAMD)_2_	O_3_	225‐250	[204]
Cu	Cu	[Cu(^s^Bu‐amd)]_2_	H_2_ plasma	150	[217]
	Cu_2_O	[Cu(^s^Bu‐amd)]_2_	H_2_ plasma: O_2_ plasma=3:1	150	[217]
	CuO	[Cu(^s^Bu‐amd)]_2_	O_2_ plasma	150	[217]
	Cu	(hfac)Cu(I)(DMB)	H_2_ plasma: O_2_ plasma=7:1	100	[220]
	Cu_2_O	(hfac)Cu(I)(DMB)	H_2_ plasma: O_2_ plasma=3:1	100	[220]
	CuO	(hfac)Cu(I)(DMB)	H_2_ plasma: O_2_ plasma=1:1	100	[220]
	Cu	Cu(I)(hfac)(tmvs)	N_2_	160	[224]
	Cu_2_O	Cu(I)(hfac)(tmvs)	H_2_O	220	[224]
	CuO	Cu(I)(hfac)(tmvs)	O_3_	260	[224]
Mo	MoO_x_ (2<x<3)	Mo(NMe_2_)_4_	H_2_O cycles: O_3_ cycles=1:0, 4:1, 1:1, 0:1	80	[240]
Sn	SnO	Sn(edpa)_2_	H_2_O	70‐300	[249]
	SnO_2_	Sn(edpa)_2_	O_2_ plasma	70‐300	[249]
	SnO	Sn(N(SiMe_3_)_2_)_2_	H_2_O	100‐250	[251]
	SnO_2_	Sn(N(SiMe_3_)_2_)_2_	O_3_	80‐200	[251]
	SnO	Sn(dmamp)_2_	H_2_O	200	[258]
	SnO_2_	Sn(dmamp)_2_	H_2_O_2_	100‐200	[258]
	SnS	Sn(dmamp)_2_	H_2_S	90‐285	[115, 290]
	SnS_2_	Sn(dmamp)_2_	H_2_S plasma	150‐240	[294]
W	W_3.32_N	W(N^t^Bu)_2_(NMe_2_)_2_	N_2_ plasma	250	[316]
	W_x_N (1.26≤x≤3.19)	W(N^t^Bu)_2_(NMe_2_)_2_	N_2_/H_2_ plasma (N_2_:H_2_=1.1‐16:1)	250	[316]
	WN	WCl_5_	N_2_/H_2_ plasma (N_2_:H_2_=1:3)	250	[317]
	W_2_N	WCl_5_	N_2_/H_2_ plasma (N_2_:H_2_=1:5)	250	[317]
	W_2.4_N	WCl_5_	N_2_/H_2_ plasma (N_2_:H_2_=1:10)	250	[317]
Ta	Ta_2_N	Ta(NMe_2_)_5_	H_2_ plasma	225	[307]
	Ta_2_N_3_	Ta(NMe_2_)_5_	N_2_/H_2_ plasma (N_2_:H_2_=1:1)	225	[307]
	TaN	TaCl_5_	NH_3_+Zn	400‐500	[309]
	Ta_3_N_5_	TaCl_5_	NH_3_	200‐500	[309]
	TaN_x_ (1≤x≤1.7)	TaF_5_	N_2_/H_2_ plasma (PN_2_/PH_2_ = 0.014‐4)	350	[310]
	TaN	TaF_5_	H_2_ plasma+NH_3_+H_2_	200	[312]
	Ta_3_N_5_	TaF_5_	H_2_ plasma+NH_3_/H_2_ plasma	200	[312]

Beyond simply selecting a single reactant to control the valence state of metal ions, more precise control of the valence states of metal ions in the films can be achieved by alternating different co‐reactants in the ALD processes [203, 204, 217, 224, 226, 235, 249, 251, 286, 307, 309, 312, 316, 317]. A representative example is the ALD of molybdenum oxides using Mo(NMe_2_)_4_, where H_2_O and O_3_ serve as co‐reactants to form MoO_2_ or MoO_3_, respectively. By adjusting the cycle ratio of H_2_O‐based to O_3_‐based ALD steps, films with intermediate valence states were systematically synthesized, as shown in Figure 5b [240]. Similarly, in the deposition of copper oxides, a two‐step process, in which metallic Cu is formed from (hfac)Cu(I)(DMB) and H_2_ plasma and subsequently oxidized by O_2_ plasma, has been proposed to tune the valence states of Cu ions. By varying the ratio of the deposition to the oxidation steps, the film composition can be precisely tuned to span the entire range from Cu to Cu_2_O and CuO [224].

### 3.4 Post‐Deposition Treatment for Tailoring Valence States

Post‐deposition treatments offer a powerful approach to tune the valence state in ALD‐grown films, particularly when the desired metastable valence states cannot be achieved under conventional ALD conditions. By decoupling the deposition and oxidation/reduction processes, such treatments enable the precise modulation of the valence states. There are several key parameters, such as temperature, atmosphere, pressure, and annealing time. These approaches are particularly effective for transition metal compounds, including V, Mn, Fe, Co, Cu, Sn, Nb, and Mo, as summarized in Table 10 [200, 205, 206, 208, 214, 216, 229, 230, 238, 239, 241, 243, 259, 261, 262, 263, 264, 265, 267, 297, 298, 305, 306].

**TABLE 10:** Representative examples of ALD processes for controlling valence states in metastable multivalent compounds through post‐deposition treatments. This table summarizes how annealing ambient, temperature, and duration correlate with changes in oxidation state and phase, summarizing general strategies for converting as‐deposited films into desired metastable valence states by thermal or chemical treatments.

Element	Material	Precursor	Reactant	Temperature (°C)	Post‐treatment	Refs.
V	V_2_O_3_	VO(acac)_2_	O_3_	200	PDA 700 °C H_2_	[259]
	V_2_O_5_	VO(acac)_2_	O_3_	200	—	[259]
	V_6_O_13_	V(NEtMe)_4_	O_3_	150	PDA 450 °C 10 Pa O_2_	[261]
	VO+VO_2_	V(NEtMe)_4_	O_3_	150	—	[263]
	VO_2_	V(NEtMe)_4_	O_3_	150	PDA 585 °C O_2_	[263]
	V_2_O_5_	V(amd)_3_	O_3_	200	—	[200]
	V_2_O_5_	V(amd)_3_	H_2_O+O_2_	200	PDA 450 °C N_2_	[200]
	V_2_O_5_	V(amd)_3_	H_2_O_2_	200	PDA 450 °C N_2_	[200]
	V_2_O_3_+VO_2_	V(amd)_3_	H_2_O_2_	200	PDA 350 °C H_2_	[200]
	VO_2_	V(amd)_3_	H_2_O_2_+H_2_	200	PDA 350 °C H_2_	[200]
	VO_2_	V(NEtMe)_4_	O_3_	150	PDA 450 °C N_2_	[265]
	V_2_O_5_	V(NEtMe)_4_	O_3_	150	PDA 450 °C Air	[265]
	VO_2_	V(NEtMe)_4_	H_2_O	150	PDA 450 °C N_2_	[265]
	V_2_O_5_	V(NEtMe)_4_	H_2_O	150	PDA 450 °C Air	[265]
	VO_2_	V(NEtMe)_4_	O_3_	150	PDA 420 °C He/3.7 Pa O_2_	[262]
	VO_2_	V(NEtMe)_4_	H_2_O	150	PDA 450 °C He/18 Pa O_2_	[262]
	V_6_O_13_	V(NEtMe)_4_	O_3_	150	PDA 550 °C He/3.7 Pa O_2_	[262]
	V_4_O_9_	V(NEtMe)_4_	H_2_O	150	PDA 356 °C Air	[262]
	V_3_O_7_	V(NEtMe)_4_	O_3_	150	PDA 560 °C He/48 Pa O_2_	[262]
	V_2_O_5_	V(NEtMe)_4_	O_3_+H_2_O	150	PDA 500 °C Air	[262]
	VO_2_	VO(O^i^Pr)_3_	H_2_O	60‐90	Ar plasma/550 °C vacuum	[264]
	V_n_O_2n‐1_	VO(O^i^Pr)_3_	H_2_O	60‐90	H_2_ plasma/550 °C vacuum	[264]
	V_2_O_5_	VO(O^i^Pr)_3_	H_2_O	135	PDA 300‐500 °C Air	[267]
	VO_2_	VO(O^i^Pr)_3_	H_2_O	135	PDA 500 °C H_2_	[267]
	VS	V(NEtMe)_4_	H_2_S	200‐300	PDA 500 °C H_2_S	[305]
Fe	Fe_2_O_3_	Fe_2_(O^t^Bu)_6_	H_2_O	130‐170	—	[229]
	Fe_3_O_4_	Fe_2_(O^t^Bu)_6_	H_2_O	130‐170	PDA 400 °C H_2_	[229]
	Fe_2_O_3_	FeCp(Cp^t^Bu)	O_2_ plasma	150‐250	—	[230]
	Fe_3_O_4_	FeCp(Cp^t^Bu)	O_2_ plasma	150‐250	PDA 385 °C H_2_	[230]
	Fe	FeCp(Cp^t^Bu)	O_2_ plasma	150‐250	PDA 430 °C H_2_	[230]
Co	Co_3_O_4_+CoO	Co_2_(CO)_8_	O_3_	50	—	[205]
	Co_3_O_4_	Co_2_(CO)_8_	O_3_	50	PDA 500 °C Air	[205]
	CoO	Co(AMD)_2_	H_2_O	180‐270	—	[206]
	Co	Co(AMD)_2_	H_2_O	180‐270	Atomic Deuterium 220 °C/Al cap 200 °C	[206]
	CoO	CoCl_2_(TMEDA)	H_2_O	225‐300	—	[208]
	Co	CoCl_2_(TMEDA)	H_2_O	225‐300	PDA 250 °C H_2_	[208]
Cu	Cu_2_O+CuO	Cu(hfac)(dmb)	O_3_	100	PDA 200‐500 °C Air	[214]
	CuO	Cu(dmap)_2_	O_3_	80‐140	—	[216]
	Cu_2_O	Cu(dmap)_2_	O_3_	80‐140	PDA 350‐700 °C N_2_	[216]
Nb	Nb_2_O_5_	Nb‐TBTEA	H_2_O	200	—	[241]
	NbO_2_	Nb‐TBTEA	H_2_O	200	PDA 1000 °C H_2_	[241]
	Nb_2_O_5_	Nb(OEt)_5_	H_2_O	170‐300	—	[243]
	Nb_2_O_5_+NbO_2_	Nb(OEt)_5_	H_2_O	170‐300	Pulsed laser annealing H_2_	[243]
Mo	MoO_3_	MoO_2_(thd)_2_	O_3_	25	PDA 450 °C Air	[238]
	MoO_2_	MoO_2_(thd)_2_	O_3_	25	PDA 450 °C H_2_	[238]
	MoO_3_	Mo(NMe_2_)_4_	O_3_	150	PDA 400 °C O_2_	[239]
	MoO_2_	Mo(NMe_2_)_4_	O_2_	150	PDA 400 °C H_2_	[239]
Sn	SnS	TDMASn	H_2_S	100	PDA 400 °C vacuum	[297]
	SnS	Sn(dmamp)_2_	H_2_S plasma	210	Sn(dmamp)_2_ feeding 270 °C	[298]
Ta	Ta_3_N_5_	Ta(N^i^Pr)(NEtMe)_3_	N_2_/H_2_ plasma	400	—	[306]
	TaN	Ta(N^i^Pr)(NEtMe)_3_	N_2_/H_2_ plasma	400	PDA 850 °C vacuum	[306]

A common approach involves thermal annealing under an oxidative or a reductive atmosphere [200, 205, 206, 208, 214, 216, 229, 230, 238, 241, 243, 259, 261, 262, 263, 264, 265, 266, 267, 297, 298, 305, 306]. Oxidative annealing increases the valence state of metal ions, whereas reductive annealing decreases that of metal ions. For instance, a mixture of VO and V_2_O_5_ grown by ALD was converted into VO_2_ through annealing in O_2_ at 585°C [263]. Similarly, Fe_2_O_3_ films prepared by ALD were reduced to Fe_3_O_4_ upon annealing in H_2_/Ar at 400°C [229]. The incorporation of plasma into the post‐deposition process can further enhance the effectiveness of the valence state control. For instance, amorphous VO_x_ films with oxidation states close to V_2_O_5_ were treated with remote H_2_ or Ar plasma at room temperature, followed by vacuum annealing at 550°C, resulting in the formation of V_n_O_2n‐1_ and VO_2_ phases, respectively [264].

In some cases, the final valence state is governed by the initial film composition and response to post‐treatment [200, 239, 240, 262, 265]. This interplay enables the highly selective synthesis of multiple phases from a single precursor system. For example, various V_2_O_3_, VO_2_, and V_2_O_5_ phases have been selectively obtained by using a V(amd)_3_ precursor with different oxidants, including O_3_, H_2_O+O_2_, H_2_O_2_, or H_2_O_2_+H_2_, followed by annealing in N_2_ or H_2_ [200]. Similarly, MoO_x_ films grown at 150°C using Mo(NMe_2_)_4_ and either O_2_ or O_3_ were post‐annealed in either H_2_ or O_2_ at 400°C, enabling compositional tuning across a wide stoichiometric range of x = 2.26–3.14 [239].

### 3.5 Noble Metals and Their Oxides

**FIGURE 6:**
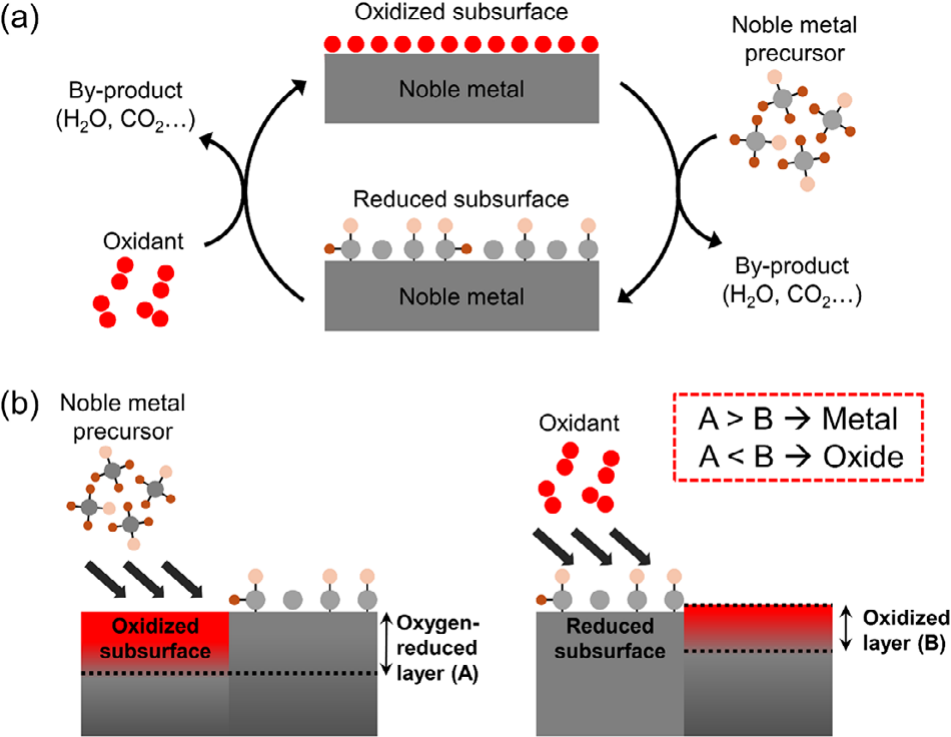
Redox‐driven ALD mechanism for noble metals and their oxides. (a) Schematic of the phase selection mechanism in the ALD processes of noble metals and their oxides. (b) Schematic illustrating how the balance between oxidation by the oxidant and reduction by the metal precursor governs the phase of the grown films.

Noble metals and their corresponding oxides can be selectively grown by ALD, enabling the controlled tuning of their valence states. As illustrated in Figure 6a, the ALD of noble metals typically employs alternating pulses of a metal precursor and an oxidizing reactant. During the injection step of a metal precursor, the metal precursor is adsorbed onto the reaction surface. The ligands of the precursor are partially removed through a reaction with pre‐adsorbed oxygen in the subsurface, reducing the subsurface. In the subsequent injection step of the oxidizing reactant, the oxidizing reactant removes the remaining ligands of the adsorbed metal precursor and re‐oxidizes the subsurface. Through repeated cycles of these two steps, film growth proceeds via alternating surface reduction and oxidation, highlighting the redox‐driven nature of noble‐metal ALD [347, 348, 349].

In ALD processes involving cyclic redox reactions, the extents of oxidation and reduction during each half‐cycle are not necessarily identical. Oxide films form when the number of oxygen atoms incorporated into the subsurface during the oxidizing reactant pulse exceeds the number of oxygen atoms removed during the subsequent metal precursor pulse. Conversely, if fewer oxygen atoms are incorporated than reduced, metallic films are obtained. The balance between the oxidation and reduction reactions is the key factor determining the final chemical state of the film, as illustrated in Figure 6b.

The redox balance in the ALD process is influenced by several parameters, including the type of metal precursor, oxidizing reactant, and growth temperature. Among these, the growth temperature is generally regarded as the most decisive parameter. As shown in Table 11, ALD processes involving noble metal elements, such as Ru, Ir, Pt, Rh, and Pd, enable the formation of oxides at relatively low temperatures [322, 323, 325, 326, 329, 330, 331, 332, 333, 341, 344, 346]. At higher temperatures, the reduction reaction induced by the precursor becomes dominant, resulting in the formation of metallic films [322, 323, 325, 326, 335, 340, 342, 345]. The critical temperature at which the transition occurs varies with the metal. For Ru and Ir, oxide formation can persist up to relative high temperatures near 270°C [318, 320, 322, 324, 325, 326, 327, 328, 334, 337], whereas for Pt, Pd, and Rh, the transition to metallic films typically occurs at significantly lower temperatures, often below 180°C [339, 341, 344, 346]. The oxidizing reactant also influences phase selection. Stronger oxidants, such as O_3_ and O_2_ plasma, are more effective than O_2_ in promoting oxide formation and can increase the upper temperature limit at which their corresponding oxides are still formed. Furthermore, the dose of the oxidizing reactant, which is determined by the pulse duration or partial pressure [318, 319, 320, 324, 325, 326, 327, 328, 329, 330, 334, 337, 339, 345], can affect the phase selection. The oxide phase is typically formed only when a sufficiently large dose of the oxidant is injected. Although the type of metal precursor has less influence on the phase selection compared with the growth temperature and oxidant, increasing the precursor dose can enhance the reduction reaction during the precursor injection, thereby favoring metal formation [318, 319]. Based on these trends, several studies have demonstrated selective phase control by tuning the dose ratio between the metal precursor and the oxidant [318, 319, 329].

**TABLE 11:** Representative examples of ALD processes for noble metals and their oxides. This table summarizes how growth temperature, oxidant strength and dose, and precursor dose control the redox balance in noble‐metal ALD and govern the transition between oxide and metallic films.

Element	Precursor	Oxidant	Phase	Temperature (°C)	Oxidant condition	References.
Ru	Ru(EtCp)_2_	O_2_	Ru	270	t_O2_/t_Ru_ <2.5	[318, 319]
	Ru(EtCp)_2_	O_2_	RuO_2_	270	t_O2_/t_Ru_ >3.3	[318, 319]
	Ru(EtCp)_2_	O_2_	RuO_2_	265	Continuous O_2_ flow	[320]
	Ru(EtCp)_2_	O_2_	Ru	300	—	[321]
	Ru(EtCp)_2_	O_2_ plasma	RuO_2_	300	—	[321]
	Ru(EtCp)_2_	O radical	Ru	300‐340	O* from O_2_/Ar with 2500W	[322]
	Ru(EtCp)_2_	O radical	RuO_2_	200‐260	O* from O_2_/Ar with 2500W	[322]
	Ru(EtCp)_2_	O_2_ plasma	Ru	375‐400	60 scm O_2_ with 75W	[323]
	Ru(EtCp)_2_	O_2_ plasma	RuO_2_	300‐350	60 scm O_2_ with 75W	[323]
	dicarbonyl‐bis(5‐methyl‐2,4‐hexanediketonato)Ru(II)	O_2_	Ru	283	t_O2_/t_Ru_: 1	[324]
	dicarbonyl‐bis(5‐methyl‐2,4‐hexanediketonato)Ru(II)	O_2_ plasma	RuO_2_	283	t_O2_/t_Ru_: 12	[324]
	Ru(DMBD)(CO)_3_	O_2_	Ru	255‐280	O_2_ 2 s	[325]
	Ru(DMBD)(CO)_3_	O_2_	RuO_2_	195‐215	O_2_ 20 s	[325]
	Ru(DMBD)(CO)_3_	O_2_ plasma	RuO_2_	195‐215	O_2_ plasma 20 s	[325]
	HD(cumene)Ru	O_2_	Ru	270‐350	200 sccm O_2_ 2 s	[326]
	HD(cumene)Ru	O_2_	RuO_2_	200	500 sccm O_2_ 20 s	[326]
	EBBDRu	O_2_	Ru	225	200 sccm O_2_ < 10 s	[327]
	EBBDRu	O_2_	RuO_2_	225	200 sccm O_2_ > 45 s	[327]
	EBCHDRu	O_2_	Ru	225	200 sccm O_2_ 1 s	[328]
	EBCHDRu	O_2_	RuO_2_	225	200 sccm O_2_ 10 – 60 s	[328]
	Ru(TMM)(CO)_3_	O_2_	RuO_2_	180	t_O2_/t_Ru_ ∼ 10	[329]
	Ru(DMPD)_2_	O_2_	RuO_2_	185	O_2_ 50 mTorr 30 s or 1 Torr 2 s	[330]
Ir	Ir(acac)_3_	O_3_	Ir	200‐225	1s	[331, 332]
	Ir(acac)_3_	O_3_	IrO_2_	165‐185	1s	[331, 332]
	Ir(acac)_3_	O_2_	Ir	300	Typically 20 s	[333]
	Ir(acac)_3_	O_3_‐H_2_	Ir	195	Typically 20 s	[333]
	Ir(acac)_3_	O_3_	IrO_2_	195	Typically 20 s	[333]
	(EtCp)Ir(COD)	O_2_	Ir	230‐290	Low O_2_/(Ar+O_2_)	[334]
	(EtCp)Ir(COD)	O_2_	IrO_2_	230‐290	High O_2_/(Ar+O_2_)	[334]
	(MeCp)Ir(COD)	O_3_‐H_2_	Ir	120‐180	—	[335]
	(MeCp)Ir(COD)	O_3_	Ir	200	—	[335]
	(MeCp)Ir(COD)	O_3_	IrO_2_	100‐180	—	[335]
	(MeCp)Ir(COD)	O_2_	Ir	225‐350	20 sccm O_2_	[336]
	TICP	O_3_	Ir	180‐250	3 – 10 s	[337]
	TICP	O_3_	IrO_2_	180‐200	30 – 60 s	[337]
	TICP	O_2_	Ir	200‐320	5 s	[338]
Pt	(MeCp)PtMe_3_	O_2_ plasma	Pt	150‐300	Short O_3_ injection	[339]
	(MeCp)PtMe_3_	O_2_ plasma	PtO_x_	100‐200	Long O_3_ injection	[339]
	(MeCp)PtMe_3_	O_2_	Pt	200‐300	—	[340]
	Pt(acac)_2_	O_3_	Pt	140‐200	6 s	[341]
	Pt(acac)_2_	O_3_	PtO_x_	120, 130	6 s	[341]
Pd	Pd(hfac)_2_	O_3_	Pd	180‐220	—	[342]
	Pd(thd)_2_	O_2_	Pd	180	—	[343]
	Pd(thd)_2_	O_3_	PdO_x_	130‐160	—	[344]
Rh	Rh(acac)_3_	O_2_	Rh	250	1‐10 s	[345]
	Rh(acac)_3_	O_3_	Rh	200‐220	0.3 s	[342]
	Rh(acac)_3_	O_3_	Rh	190	6‐20 s	[346]
	Rh(acac)_3_	O_3_	Rh_2_O_3_	160‐180	6‐20 s	[346]

Owing to the inherent instability of noble metal oxides, even when oxide films are formed at low temperatures, oxides can be readily reduced to their metallic state upon exposure to reducing agents, such as hydrogen [344, 350, 351] or ethylcyclohexane [352]. In fact, a low‐temperature ALD strategy has been reported in which the precursors O_3_ and H_2_ are introduced sequentially within a single cycle to efficiently deposit metallic films [344, 350, 351].

## 4 Conclusions and Outlook

Metastable phases present a powerful avenue for expanding the novel functionality of thin films and unlocking the properties essential for next‐generation electronics, catalysis, and energy‐related applications. However, the inherent challenges in the synthesis of these phases continue to hinder their broader application. Owing to its precise atomic control and excellent conformality, ALD has attracted interest across industries and has emerged as a tool for stabilizing such phases. In particular, as the need for high‐performance materials compatible with 3D integration grows, achieving metastable phases via ALD becomes a critical strategy. Although a low temperature window may hinder the facile synthesis of metastable phases, recent advances in process design and novel mechanistic innovations have steadily overcome these limitations.

This review outlines the recent strategies for realizing metastable phases via ALD. Metastable phases are categorized into two main classes: the selection of polymorphic phases, and the tuning of valence states. For polymorph control, strategies, such as controlling the deposition temperature, substrate‐induced lattice matching, grain‐size refinement, and chemical modulation by incorporating heteroelements, have been introduced. Strategies, such as the control of the deposition temperature, the design and selection of precursors and co‐reactants, and post‐deposition treatments, have been systematically exploited to tune the valence state. Such strategies have extended ALD to the synthesis of diverse metastable materials, such as oxides, chalcogenides, nitrides, and even elemental metals. This demonstrated that ALD is a highly versatile tool for stabilizing metastable phases.

Important opportunities remain. The process window for synthesizing metastable phases remains narrow, and expanding this accessible regime is critical for a broader impact. For instance, the dependence on specific substrates to stabilize certain polymorphs has significant limitations. Approaches that decouple metastability from substrate selection, such as the recent use of sacrificial layers [92], highlight promising directions for addressing this limitation.

To further unlock the synthesis of metastable phases by ALD, it is imperative to employ a rational precursor design tailored to specific reaction pathways and develop a mechanistic understanding of surface reactions via in situ diagnostics. For example, synchrotron‐based in situ X‐ray measurements during ZnO ALD have revealed the early‐stage evolution of nucleation and film coalescence [353]. Likewise, cycle‐resolved in vacuo XPS during Pt ALD has captured transient surface reaction states that define the initial growth chemistry [354]. These real‐time measurements including in situ X‐ray reflectivity, in vacuo XPS, and grazing‐incident wide‐angle X‐ray scattering, reveal transient surface configurations and kinetic constraints that are inaccessible ex situ, providing mechanistic insight into how transient ordering and surface reaction pathways can bias the system toward the stabilization of metastable phases.

Looking ahead, we anticipate that data‐driven approaches will play an increasingly central role. The integration of artificial‐intelligence‐ and machine‐learning‐assisted metastability prediction, tailored to ALD‐specific variables such as precursor chemistry, reactant type, substrate, and temperature window, will enable the identification of kinetic stabilization pathways and provide guidance for experimentally targeting phases that have not yet been accessed by ALD. By combining first‐principles calculations, high‐throughput simulations, and experimentally derived databases with these models, it should become possible to rapidly screen chemistries and process conditions, thereby accelerating the discovery of metastable phases while minimizing costly trial‐and‐error in the laboratory.

These advances will open opportunities not only within the material families already explored, but also in chemistries that have so far remained underrepresented in ALD. For instance, metastable fluorides and phosphides, which often exhibit rich polymorphism and diverse valence states, have yet to be extensively studied in the context of ALD. These materials may present unique opportunities for tailoring optical, electronic, or ionic transport properties, especially in applications such as batteries, catalysis, and optoelectronics. Identifying suitable precursors and reaction schemes for such chemistries represents an important direction for future research. Van der Waals materials represent another frontier where metastable phases may play a pivotal role. The atomically thin nature and weak interlayer interactions of these systems often result in energy landscapes with multiple competing minima, making them ideal candidates for metastable phase stabilization via surface‐controlled processes such as ALD.

With the increasing demand for innovative materials, atomic layer deposition is expected to evolve from a mere deposition technique to a versatile platform for metastable material design, enabling access to exotic phases that surpass thermodynamic constraints and redefining the limits of material discovery. Ultimately, the continued refinement of these strategies will accelerate the integration of metastable phases into next‐generation functional materials, contributing to progress across a wide range of applications.

## Conflicts of Interest

The authors declare no conflict of interest.

## Data Availability

The authors have nothing to report.
